# A Myeloperoxidase-Containing Complex Regulates Neutrophil Elastase Release and Actin Dynamics during NETosis

**DOI:** 10.1016/j.celrep.2014.06.044

**Published:** 2014-07-24

**Authors:** Kathleen D. Metzler, Christian Goosmann, Aleksandra Lubojemska, Arturo Zychlinsky, Venizelos Papayannopoulos

**Affiliations:** 1 Department of Cellular Microbiology, Max Planck Institute for Infection Biology, Berlin 10117, Germany; 2 Division of Molecular Immunology, Medical Research Council National Institute for Medical Research, London NW7 1AA, UK

## Abstract

Neutrophils contain granules loaded with antimicrobial proteins and are regarded as impermeable organelles that deliver cargo via membrane fusion. However, during the formation of neutrophil extracellular traps (NETs), neutrophil elastase (NE) translocates from the granules to the nucleus via an unknown mechanism that does not involve membrane fusion and requires reactive oxygen species (ROS). Here, we show that the ROS triggers the dissociation of NE from a membrane-associated complex into the cytosol and activates its proteolytic activity in a myeloperoxidase (MPO)-dependent manner. In the cytosol, NE first binds and degrades F-actin to arrest actin dynamics and subsequently translocates to the nucleus. The complex is an example of an oxidative signaling scaffold that enables ROS and antimicrobial proteins to regulate neutrophil responses. Furthermore, granules contain protein machinery that transports and delivers cargo across membranes independently of membrane fusion.

## Highlights

• A complex of antimicrobials drives neutrophil elastase to the nucleus during NETosis
• The “azurosome” complex mediates protein release across intact membranes
• Myeloperoxidase is required for neutrophil elastase release
• Neutrophil elastase degrades F-actin and arrests actin dynamics

## Graphical Abstract

**Figure:**
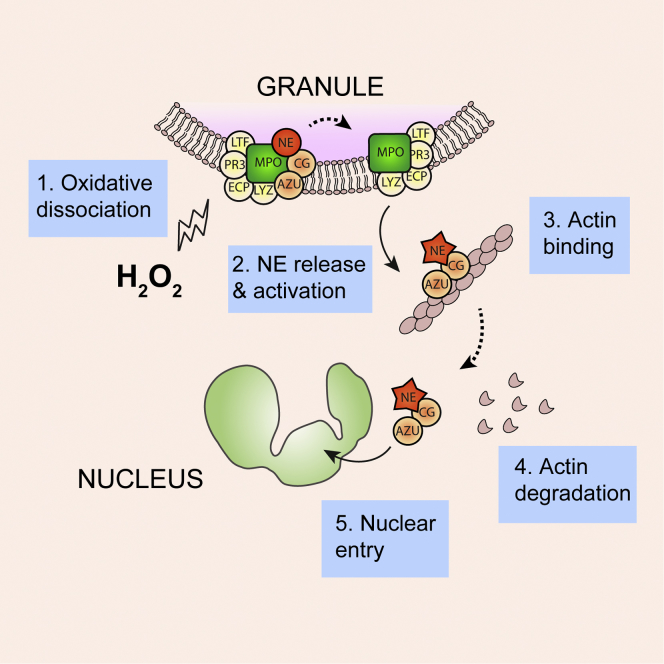

## Teaser

Neutrophils combat pathogens by releasing decondensed web-like chromatin structures called neutrophil extracellular traps (NETs). During NET formation, a protease called neutrophil elastase (NE), which is stored in subcellular membrane compartments called granules, translocates to the nucleus, cleaving histones to promote chromatin decondensation. Metzler et al. show that a complex of antimicrobial proteins named “azurosome” mediates NE release in response to reactive oxygen species independently of membrane fusion and without disrupting membrane integrity, while activating NE to degrade F-actin and arrest actin dynamics.

## Introduction

Neutrophils are the foot soldiers of the innate immune system as they are plentiful, short-lived, and armed with antimicrobial effector strategies. They are the first immune cells to arrive at a site of infection and are ready to respond, carrying presynthesized antimicrobial effectors and the enzymes needed to mount an intense burst of reactive oxygen species (ROS) (Amulic et al., 2012). Antimicrobial effectors are synthesized during neutrophil development and are stored in specialized membrane-bound vesicles called granules. Granules contain different cargo depending on when they were synthesized. This results in a continuum of granule contents that are classified into four categories: secretory vesicles and azurophilic, specific, and gelatinase granules (Borregaard, 2010).

Granule membranes are regarded as impermeable barriers that allow for delivery of their cargo through membrane fusion. Neutrophils ingest and kill microbes intracellularly through phagocytosis. During this process, microbes are enclosed in a membrane compartment known as the phagosome, where exposure to ROS and antimicrobial effectors eliminates pathogens. The antimicrobial load of granules is delivered to the phagosome by fusion of the granule and phagosomal membranes. In addition, granules can fuse with the plasma membrane to release granule cargo extracellularly through degranulation.

In contrast to this classical view, an antimicrobial strategy that involves some unconventional cell biology was recently uncovered. Neutrophils were shown to release web-like structures known as neutrophil extracellular traps (NETs) that ensnare and kill a variety of microbes. NETs are composed of decondensed chromatin and a subset of granule and cytoplasmic proteins (Brinkmann et al., 2004). Patients and animals carrying mutations in the genes required for NET formation are more susceptible to infections (Brinkmann and Zychlinsky, 2012). On the other hand, unregulated NET release or lack of NET degradation is linked to several diseases, including cystic fibrosis, preeclampsia, autoimmunity, and vascular diseases (Garcia-Romo et al., 2011; Hakkim et al., 2010; Kessenbrock et al., 2009; Khandpur et al., 2013; Lande et al., 2011; Papayannopoulos et al., 2011; Villanueva et al., 2011). Therefore, it is critical to understand the mechanisms that regulate NET formation.

NETs form in response to specific stimuli through a unique form of cell death called “NETosis.” The nuclear material expands while chromatin decondenses and the nuclear envelope disintegrates. The cytoplasmic membrane ruptures, liberating the NETs (Fuchs et al., 2007). A fraction of neutrophils have also been reported to release NETs without dying, leaving behind cytoplasts that continue to ingest microbes (Pilsczek et al., 2010; Yipp et al., 2012).

Α factor that is known to be critical for NET formation is neutrophil elastase (NE) (Papayannopoulos et al., 2010). This serine protease is stored in azurophilic granules and contributes to antimicrobial activity in the phagosome. During NET formation, NE translocates from the granules to the nucleus and partially cleaves histones to promote chromatin decondensation (Papayannopoulos et al., 2010). The mechanism of NE release from azurophilic granules remains unknown and does not involve membrane fusion.

ROS are crucial for effective antimicrobial responses. Patients with chronic granulomatous disease (CGD), who are deficient in NADPH oxidase activity, and individuals who are completely deficient in myeloperoxidase (ΔMPO; Figure S1A) are susceptible to opportunistic infections, particularly to fungal pathogens (Nauseef, 2007). Neutrophils from these patients fail to form NETs when stimulated with physiological NET stimuli such as fungi or the ROS agonist phorbol myristate acetate (PMA) (Fuchs et al., 2007; Metzler et al., 2011).

Upon stimulation, neutrophils rapidly activate the NADPH oxidase to generate superoxide, a highly reactive molecule that dismutates to hydrogen peroxide (H_2_O_2_) (Winterbourn and Kettle, 2013). H_2_O_2_ is consumed by MPO to produce hypochlorous acid (HOCl) and other oxidants. MPO is also required for NET formation, as shown in donors with complete MPO deficiency, but its role remains unclear (Metzler et al., 2011). Although ROS are cytotoxic, they are also important signaling mediators that regulate protein function via the oxidation of specific amino acid residues (Nathan, 2003; Tonks, 2005; Winterbourn, 2008). However, since ROS are highly reactive, short-lived molecules, it is unclear how they are able to produce specific cellular responses. In particular, during NET formation, it is not known whether and how ROS regulate the selective translocation of NE from the granules to the nucleus. Furthermore, as the nucleus begins to decondense during NET formation, neutrophil chemotaxis is arrested through an unknown mechanism.

Using primary human neutrophils and proteins purified from healthy individuals and patient donors, we show that NE translocation involves a mechanism that does not require membrane fusion and regulates protease activation and actin dynamics.

## Results

### ROS and MPO Are Required for NE Release from Granules

**Figure 1:**
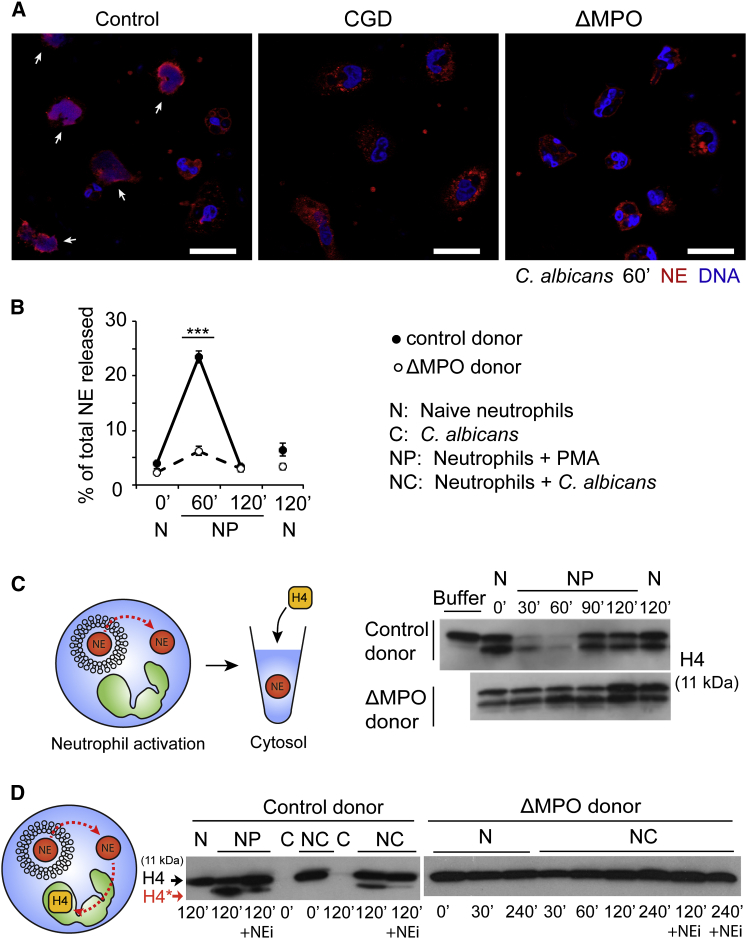
ROS and MPO Are Required for NE Translocation during NETosis (A) Single confocal microscopy images of neutrophils from control, CGD, and ΔMPO donors. The neutrophils were stimulated for 60 min with *C. albicans*, immunolabeled for NE (red), and stained for DNA (Draq5, blue). Arrows indicate nuclear NE. Scale bars, 20 μm. (B) NE release into the cytosol during NETosis measured by ELISA in cytosolic extracts derived from naive neutrophils alone (N) or PMA-activated neutrophils (NP) from control and ΔMPO donors. NE in the cytosol normalized to the total amount of NE in the cytoplasmic extract of each sample at time 0. Error bars indicate SD in triplicate samples; ^∗∗∗^p < 0.001 between control and ΔMPO samples at 60 min. Cytoplasmic extracts were made by nitrogen cavitation, without detergent, to keep the granule membranes intact. Cytosolic extracts were made by ultracentrifugation of cytoplasmic extracts. (C) Immunoblot of the degradation of exogenous histone H4 by cytoplasmic extract from naive neutrophils alone (N) or PMA-activated (NP) control and ΔMPO neutrophils. The cells were activated for the indicated time durations and H4 was incubated with the cytoplasmic extracts for 3 hr. (D) Immunoblot against endogenous histone H4 in total cell lysates of naive neutrophils alone (N) from control or ΔMPO donors. Naive neutrophils (N) or stimulated with PMA (NP) or *C. albicans* (NC) for the indicated durations in the presence (+NEi) or absence of NEi. Full-length (H4, arrow) and proteolytically processed H4 (H4^∗^, red arrow). C, *C. albicans* alone. See also Figure S1.

Since ROS production precedes NE translocation to the nucleus, we tested whether ROS and MPO are required for this process. In contrast to neutrophils from healthy “control” donors, in neutrophils derived from CGD and ΔMPO donors stimulated with *Candida albicans* (Figure 1A) or PMA (Figure S1B), NE failed to translocate to the nucleus and remained in granules.

We examined whether NE is first released from the granules into the cytosol. We stimulated neutrophils with PMA, lysed them at different time points, and isolated cytoplasm containing the soluble cytosol and granules. We obtained cytosol, which contains only the released soluble proteins, by ultracentrifugation of cytoplasm to remove granules, and monitored the presence of NE in these subcellular fractions by ELISA. NE was detected transiently in the cytosol of control neutrophils 60 min after activation (Figure 1B). Consistent with previous observations (Papayannopoulos et al., 2010), NE disappeared from the cytosol 120 min after activation, as it translocated to the nucleus. In contrast, in ΔMPO neutrophils, NE was not detected in the cytosol.

NE proteolytic activity in the cytosol from naive and activated neutrophils was also detected by adding purified recombinant histone H4 to these fractions. H4 is the relevant NE substrate during NETosis. Background partial H4 cleavage was detected in cytosol from naive neutrophils, which may be due to cytosolic proteases. Notably, H4 was completely degraded when incubated with cytosol from control neutrophils stimulated with PMA for 30 min (Figure 1C). This is consistent with the presence of active NE in the cytosol, since we previously showed that this protease degrades soluble H4 processively (Papayannopoulos et al., 2010). The peak of processive H4 degradation coincided with the highest cytosolic NE concentration detected by ELISA (Figure 1C). Recombinant H4 degradation was blocked by the small-molecule, cell-permeable NE inhibitor (NEi) GW311616A (Macdonald et al., 2001), but not by an inhibitor of the related azurophilic granule protease, cathepsin G (CGi; Figure S1E), indicating that H4 was degraded by active NE in the cytosol. In contrast, only background protease activity was detected in the cytosol of ΔMPO neutrophils, suggesting that MPO is required for the release of proteolytically active NE from the granules into the cytosol during NET formation.

Since the final destination of NE during NETosis is the nucleus, where it targets core histones, we examined the degradation of endogenous neutrophil nuclear H4 in activated control and ΔMPO neutrophils. Histone H4 was not cleaved in ΔMPO neutrophils stimulated with *C. albicans* (Figure 1D) or ΔMPO and CGD neutrophils stimulated with PMA (Figures S1C and S1D). Thus, ROS and MPO are required for the release of NE to the cytosol and its subsequent translocation to the nucleus during NETosis. Importantly, the route of NE translocation via the cytosol hinted that the translocation was driven by a novel MPO-dependent mechanism that does not involve membrane fusion.

We also examined whether this mechanism is implicated in the delivery of NE to the phagosome, which involves membrane fusion. Notably, NE cleaves bacterial virulence factors such as the *Shigella flexneri* IpaB protein inside the phagosome, preventing microbial escape from the phagosome (Weinrauch et al., 2002). Therefore, we tested whether MPO is required for NE function in the phagosome by incubating neutrophils with *S. flexneri* and examining the cleavage of the phagocytosed IpaB. IpaB was cleaved equally well by control neutrophils in the absence and presence of a pharmacological MPO inhibitor (ABAH; Figure S1F). Furthermore, neutrophils derived from a ΔMPO donor degraded IpaB with comparable efficiency. As expected, IpaB degradation was prevented in control and ΔMPO neutrophils when NE activity was inhibited pharmacologically by NEi (Macdonald et al., 2001). Thus, this mechanism for NE release appears to be specific to NET formation and not phagocytosis.

### Azurophilic Granules Contain the Machinery for NE Release

**Figure 2:**
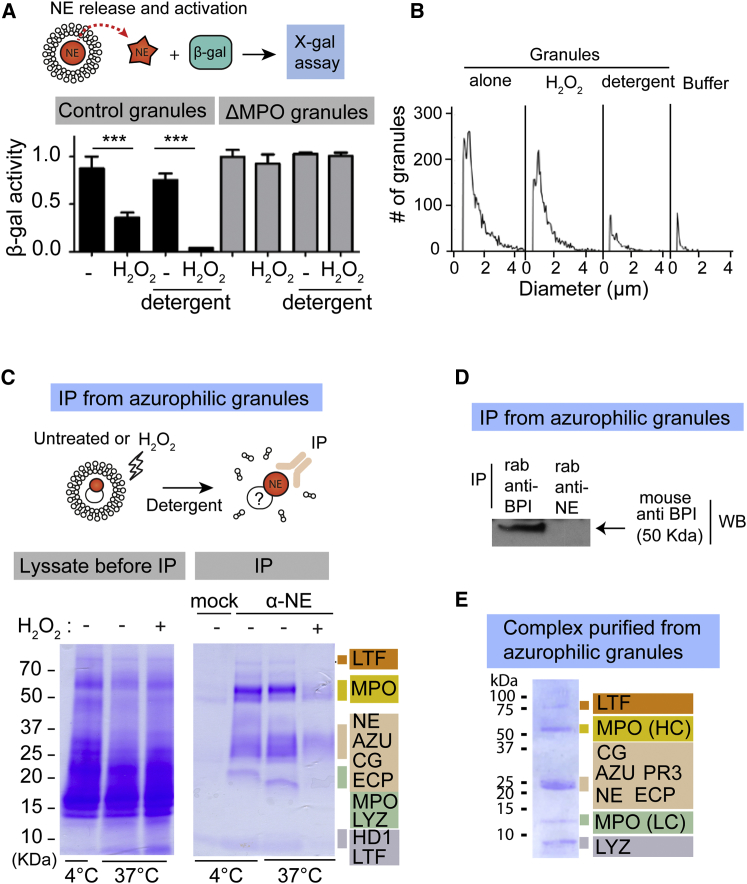
Azurophilic Granules Contain the Azurosome Complex (A) β-galactosidase activity against X-gal after incubation with azurophilic granules from a control and a ΔMPO donor in the absence or presence of H_2_O_2_ and/or detergent. Error bars indicate SD in triplicate samples; ^∗∗∗^p < 0.001 between the indicated samples. (B) CASY impedance cell counter analysis of azurophilic granules, either untreated or treated with H_2_O_2_ or with detergent. (C) Coomassie stain of azurophilic granule lysates before immunoprecipitation (IP, left), or proteins immunoprecipitated with anti-NE (IP α-NE) or control anti-MMP9 antibody (mock) from isolated azurophilic granules untreated or treated with hydrogen peroxide (H_2_O_2_) (IP, right). LTF, lactoferrin; MPO, myeloperoxidase; AZU, azurocidin; CG, cathepsin G; ECP, eosinophil cationic protein; LYZ, lysozyme; HD1, defensin-1. (D) IP of solubilized azurophilic granule extract with rabbit anti-BPI or rabbit anti-NE, followed by immunoblotting with a mouse anti-BPI antibody. (E) Coomassie stain of purified azurosome complex (fractions 19–22 from Figure S3B, pooled and concentrated). PR3, proteinase 3. See also Figure S2.

Next, we investigated whether the factors that mediate NE release are contained in azurophilic granules. We isolated intact azurophilic granules by nitrogen cavitation and discontinuous Percoll density gradient centrifugation, which separates this granule subtype from other neutrophil granules and cytosol (Kjeldsen et al., 1994). To detect release of NE, we incubated granules with exogenous β-galactosidase and monitored its degradation by loss of β-galactosidase activity. To avoid variations in protease content between different granule preparations, we normalized the concentration of granules based on the content of NE and CG as measured by ELISA and immunoblotting. First, we tested whether H_2_O_2_ was sufficient to trigger NE release in this system. Several lines of evidence suggest that H_2_O_2_, the substrate of MPO, is a key ROS intermediate in NET formation, since it is sufficient to stimulate NET formation in neutrophils (Fuchs et al., 2007) and in a cell-free assay where neutrophil nuclei are incubated with cytoplasmic extracts containing azurophilic granules in vitro (Figure S2A; Papayannopoulos et al., 2010). Moreover, catalase, which consumes H_2_O_2_, blocks NET formation (Fuchs et al., 2007; Parker et al., 2012). Azurophilic granules from control donors degraded β-galactosidase upon treatment with H_2_O_2_, indicating that active proteases were released and gained access to the substrate in the absence of detergent (Figures 2A and S2B). NEi and CGi together, but not individually, decreased β-galactosidase degradation, indicating the release of multiple active proteases (Figure S2C). Surprisingly, H_2_O_2_ did not disrupt the overall integrity of the granules, as reflected by the conservation of the granule signature in a CASY impedance counter, which measures membrane integrity by the exclusion of electrical current (Figure 2B). This observation suggested that the mechanism of NE release does not involve the dissolution of granule membranes, but rather a novel means of release from intact granules. Moreover, H_2_O_2_ failed to induce β-galactosidase degradation in ΔMPO granules, suggesting that MPO is required for NE release (Figures 2A and S2B).

To examine whether H_2_O_2_ plays a role in activating proteases in this assay independently of their release, we tested β-galactosidase degradation after dissolving granule membranes with detergent to expose the substrate to the proteases. In control granules, addition of detergent did not induce β-galactosidase degradation, but proteolytic activity required H_2_O_2_ even in the absence of membranes (Figures 2A and S2B). In contrast, H_2_O_2_ treatment failed to activate proteases in ΔMPO granules treated with detergent. Therefore, the factors that drive protease activation and release in response to H_2_O_2_ are localized in azurophilic granules, and MPO is critical for both processes.

### Isolation and Identification of an Azurophilic Granule Complex

To identify the factors that mediate NE release and activation, we probed for NE-binding partners in azurophilic granules by immunoprecipitation. We isolated azurophilic granules from control neutrophils, solubilized them with detergent, and immunoprecipitated proteins with an antibody against NE or a control mock antibody against matrix metalloproteinase 9 (MMP9), a protein that is stored in gelatinase granules. Anti-NE, but not the control antibody, selectively coimmunoprecipitated a granule protein complex containing MPO, azurocidin (AZU), CG, eosinophil cationic protein (ECP), defensin-1 (HD1), lysozyme (LYZ), and lactoferrin (LTF) (Figure 2C). LTF is primarily a specific granule protein, but it has also been found in azurophilic granules (Lominadze et al., 2005). Western blot analysis confirmed the specific immunoprecipitation of several of these proteins (see Figures 4B–4D). To further confirm the specificity of the immunoprecipitation, we immunoblotted against an azurophilic granule protein that was not immunoprecipitated. The bactericidal/permeability-increasing protein (BPI) was detected only upon immunoprecipitation with an antibody against BPI, and not with an anti-NE antibody (Figure 2D).

Interestingly, treatment of intact granules with H_2_O_2_ prior to solubilization and immunoprecipitation led to the dissociation of this complex, as significantly less protein was coimmunoprecipitated (Figure 2C). In order to investigate the effects of oxidation on the complex, we isolated azurophilic granules from peripheral blood neutrophils of healthy human donors and purified the complex by size-exclusion chromatography, probing the fractions for NE and MPO (Figures S3A and S3B). The complex eluted at a higher molecular weight than purified MPO (Figure S3B) and contained the same proteins that coprecipitated with NE as detected by mass spectrometry (Figure 2E). We also identified proteinase 3 (PR3), a related azurophilic granule protease with high homology to NE (Korkmaz et al., 2008), in the purified complex. NE and MPO are present in the complex at a ratio of 2:1. Similarly to the immunoprecipitated complex (Figure 2C), the purified complex dissociated when pretreated with H_2_O_2_, as we did not detect these proteins in the complex-containing fractions by mass spectrometry, ELISA, or enzymatic activity (Figures S3C–S3E). This observation hinted that H_2_O_2_ may regulate the function of this azurophilic granule complex by modulating the association of its components. To facilitate the nomenclature, we refer to this azurophilic granule complex as the “azurosome.”

### NE and MPO Localize to the Membrane in a Subset of Azurophilic Granules

**Figure 3:**
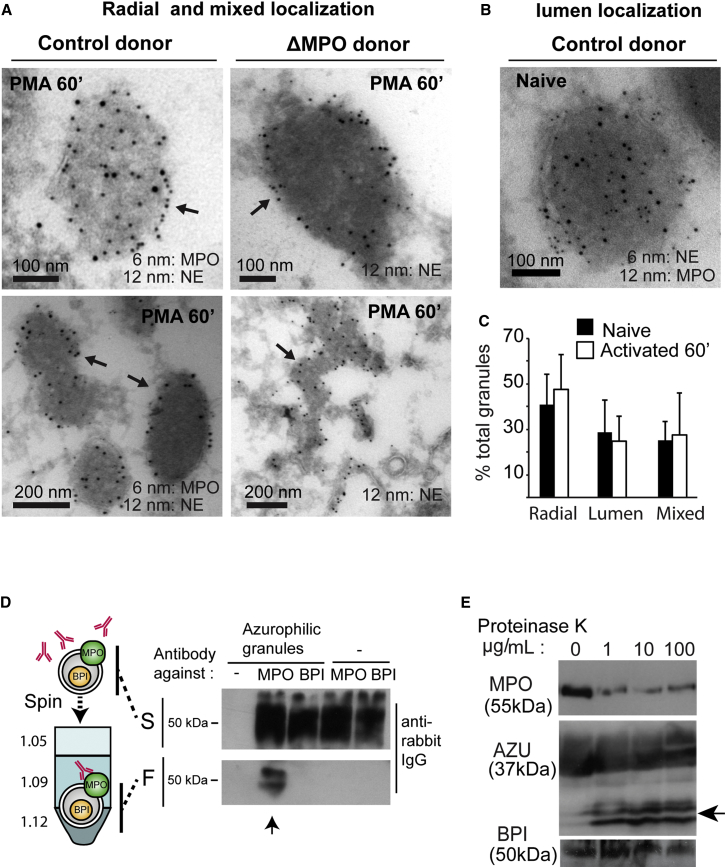
NE and MPO Localize to the Membrane in a Subpopulation of Granules (A) Immunoelectron micrographs of granules from control or ΔMPO neutrophils, naive or stimulated for 60 min with PMA and labeled with antibodies against MPO and NE coupled to 6 and 12 nm particles. Arrows indicate the membrane localization of NE and MPO. (B) Representative immunoelectron micrographs of azurophilic granules exhibiting localization of MPO and NE in the granule lumen. Control neutrophils were either left untreated or stimulated with PMA for 60 min before fixing and immunogold labeling for MPO and NE. (C) Average distribution per single cell of granules with the indicated MPO and NE localization in electron micrographs from eight naive and 13 PMA activated neutrophils; 114 and 153 granules, respectively, were counted. Error bars indicate SD within each granule group. Nonparametric ANOVA for median differences, p = 0.003. (D) Azurophilic granules incubated with rabbit IgG against MPO or BPI, fractionated by centrifugation over a discontinuous Percoll gradient of 1.05, 1.09, and 1.12 g/ml density. The soluble (S) top layer and the 1.09/1.12 interface that contains the intact azurophilic granules were collected to detect primary antibodies by SDS-PAGE electrophoresis and western immunoblotting with anti-rabbit IgG. Arrows point to MPO antibody that cofractionates with azurophilic granules. (E) Intact azurophilic granules from naive neutrophils alone or treated with proteinase K (0, 1, 10, and 100 μg/ml) and immunoblotted for MPO, AZU, and BPI. Arrow indicates the cleavage product of AZU.

To investigate the localization of the complex in neutrophils, we labeled MPO and NE with immunogold and performed transmission electron microscopy. We found three subsets of granules in naive and activated neutrophils of control and MPO-deficient neutrophils. In one subset, NE and MPO localized to the granule membrane in a radial pattern (Figure 3A, arrows). In the second subset of granules, NE and MPO were predominantly in the lumen (Figure 3B). In a third subpopulation, MPO and NE localized to both the membrane and the lumen (mixed). Our observations are consistent with similar findings in promyelocytes (Egesten et al., 1994) and confirm the heterogeneity of azurophilic granules (Borregaard, 2010). Quantitation of electron micrographs showed that MPO and NE were localized exclusively in the membrane in 50% of labeled granules and exclusively in the lumen in 25%. In the remaining 25%, the proteins were localized in both the membrane and the lumen (Figure 3C). These data are consistent with the idea that azurosome components localize in the membrane in a subset of azurophilic granules, but they do not constitute a quantitative assessment of protein association and abundance.

To determine whether the azurosome is exposed on granule membranes, we incubated isolated native azurophilic granules with an antibody against MPO or a control antibody against BPI, a protein that is not found in the azurosome (Figure 2D) and is not expected to be on the membrane. We centrifuged the mixture of granules and antibodies over a discontinuous Percoll gradient and isolated the intact azurophilic granules from the appropriate gradient fraction. Only the antibody against MPO was detected in the azurophilic granule fraction, and the antibody against BPI did not cosediment, confirming that the granule membranes were intact and undamaged, shielding BPI from antibody recognition (Figure 3D). Neither of the two antibodies sedimented in the absence of granules. These results indicated that MPO is exposed on the surface of azurophilic granules and is accessible to antibodies added externally.

Consistently, treatment of azurophilic granules from naive control neutrophils with proteinase K in the absence of detergent partially degraded MPO and AZU, indicating that in naive neutrophils, a fraction of MPO and AZU are exposed at the surface of granules (Figure 3E). AZU was better protected than MPO, suggesting that the former may be less exposed. BPI was completely protected from degradation, corroborating that the granules were intact and the exposure of MPO and AZU was not due to damaged granule membranes. This was further confirmed by impedance measurement of membrane integrity (not shown).

Together, these data suggest that naive neutrophils contain a subset of azurophilic granules that harbor the azurosome on their membranes and are poised to release proteases upon oxidative stimulation. The association with membranes does not seem to require MPO, since NE localizes to granule membranes in both control and ΔMPO neutrophils (Figure 3A). Rather, MPO is required for the ability of the complex to release proteins across membranes.

### H_2_O_2_ Triggers the Dissociation of Granule Proteases from the Complex

**Figure 4:**
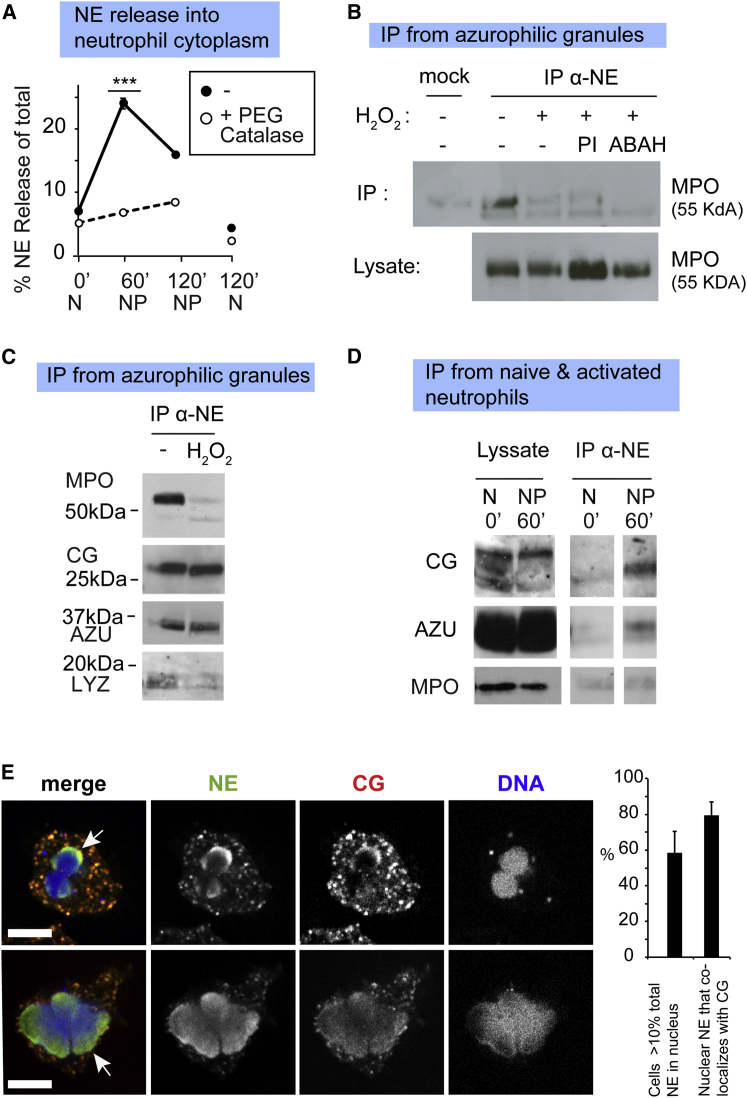
H_2_O_2_ Drives the Dissociation of the Azurosome (A) NE release into the cytosol by untreated or PEG-catalase-treated neutrophils at the indicated time points, measured by proteolytic activity against a chromogenic NE substrate, since catalase interferes with the peroxide-based ELISA readout. Data were normalized to the amount of NE in the cytoplasm of each sample at the start of the time course. Error bars indicate SD in triplicate samples; ^∗∗∗^p < 0.001 between 60′ PMA untreated versus catalase-treated triplicates. (B) Immunoprecipitation from a control azurophilic granule lysate with α-NE or α-MMP9 antibody (mock), followed by immunoblotting against MPO. Granules were left untreated or treated with H_2_O_2_ in the absence or presence of the protease inhibitors (PIs) NEi, CGi, PMSF and Roche cocktail, or MPO inhibitor (ABAH). The input lysate prior to immunoprecipitation is shown in the bottom lane. (C) Immunoprecipitation from a control azurophilic granule lysate, untreated or treated with H_2_O_2,_ using a α-NE antibody, followed by immunoblotting against MPO, CG, AZU, or LYZ. (D) Immunoprecipitation with anti-NE (IP α-NE) from cytoplasmic neutrophil lysate of naive (N, 0′) or PMA-activated (NP, 60′) control neutrophils. Left lanes: total protein in the cytoplasmic lysate before immunoprecipitation. Right lanes: proteins immunoprecipitated with an α-NE antibody from cytoplasm and immunoblotted with antibodies against CG, AZU, or MPO. Images are from the same exposed blot, but were separated to remove irrelevant lanes. (E) Single confocal microscopy sections of control neutrophils stimulated with PMA for 60 min and immunolabeled for CG (red) and NE (green). The nucleus was labeled with the DNA stain Draq5 (blue). Arrows indicate nuclear NE and CG. Upper panels: a neutrophil during the early stage of NETosis. Lower panels: a neutrophil in a later stage, exhibiting a large decondensed nucleus. Scale bars, 10 μm. Right: quantitation of the percentage of neutrophils that contained more than 10% of total NE in the nucleus and the percentage of nuclear NE that colocalized with CG. Error bars indicate SD in duplicate samples.

The results shown in Figure 2C suggested that oxidants may regulate NE release from the granule by modulating the newly identified membrane-associated complex. To address whether H_2_O_2_ is required for NE release during NETosis in neutrophils, we tested whether depleting intracellular H_2_O_2_ with PEG-catalase, which is taken up by the cells and consumes H_2_O_2_, would block NE release into the cytosol. PEG-catalase completely blocked NE release, indicating that H_2_O_2_ regulates this process and is required for NETosis (Figure 4A).

Next, we investigated how H_2_O_2_ triggers NE release. We previously found that during NETosis, NE translocates to the nucleus while MPO remains in the granules (Papayannopoulos et al., 2010). The association of NE with MPO in granules of naive neutrophils suggested that H_2_O_2_ stimulation may trigger their dissociation. Indeed, by immunoprecipitating the azurosome with an anti-NE antibody and immunoblotting, we found that in azurophilic granules treated with H_2_O_2_, MPO dissociated from NE (Figure 4B). Importantly, MPO dissociation did not require NE activity, since it was not blocked by a cocktail of protease inhibitors (PIs) against NE, CG, and other proteases (Figure 4B). The disappearance of immunoprecipitated MPO was not due to degradation or lack of recognition by the anti-MPO antibody, since MPO was detected in the lysate prior to immunoprecipitation in all samples. In addition, MPO dissociated from NE when the enzymatic activity of MPO was blocked pharmacologically with ABAH. Since ABAH does not completely suppress MPO activity (Figure S4A), we tested the ability of HOCl, the main product of MPO in these reactions, to drive complex dissociation. NE and MPO remained bound upon treatment of azurophilic granules with HOCl (Figure S4B), indicating that H_2_O_2_ likely is sufficient to drive dissociation. However, one cannot rule out the possibility that dissociation is driven by other MPO oxidative products.

We also examined the dissociation of other azurosome proteins after treatment of azurophilic granules with H_2_O_2_ and found that NE dissociates from MPO and LYZ, but remains bound to CG and AZU (Figure 4C). These data further confirm that the loss of MPO is due to dissociation from the complex, and not to inefficient immunoprecipitation, as these other proteases are pulled down with comparable efficiency. Consistent with these in vitro observations, we found a similar molecular pattern for release in activated neutrophils isolated from human control donors, where a complex of NE, CG, and AZU, but not MPO, coimmunoprecipitated from the cytosol at 60 min poststimulation (Figure 4D). Moreover, NE and CG translocated to the nucleus simultaneously in neutrophils activated with PMA (Figure 4E) or *C. albicans* (Figure S4C). In contrast, MPO and PR3 remained in granules during this early phase of NET formation (Papayannopoulos et al., 2010). Therefore, during NET formation, H_2_O_2_ triggers the dissociation of the complex with MPO remaining in the granules and a protease subcomplex selectively released into the cytoplasm.

### The Azurosome Mediates MPO-Dependent Protein Release from Intact Granules

To address the role of MPO in NE release, we tested the ability of purified complex from healthy control and ΔMPO human donors to release the contents of calcein-loaded synthetic liposomes in the absence of other membrane proteins. Inside liposomes, calcein is packed at high concentrations that quench its fluorescence. Calcein release alleviates quenching, causing an increase in calcein fluorescence.

**Figure 5:**
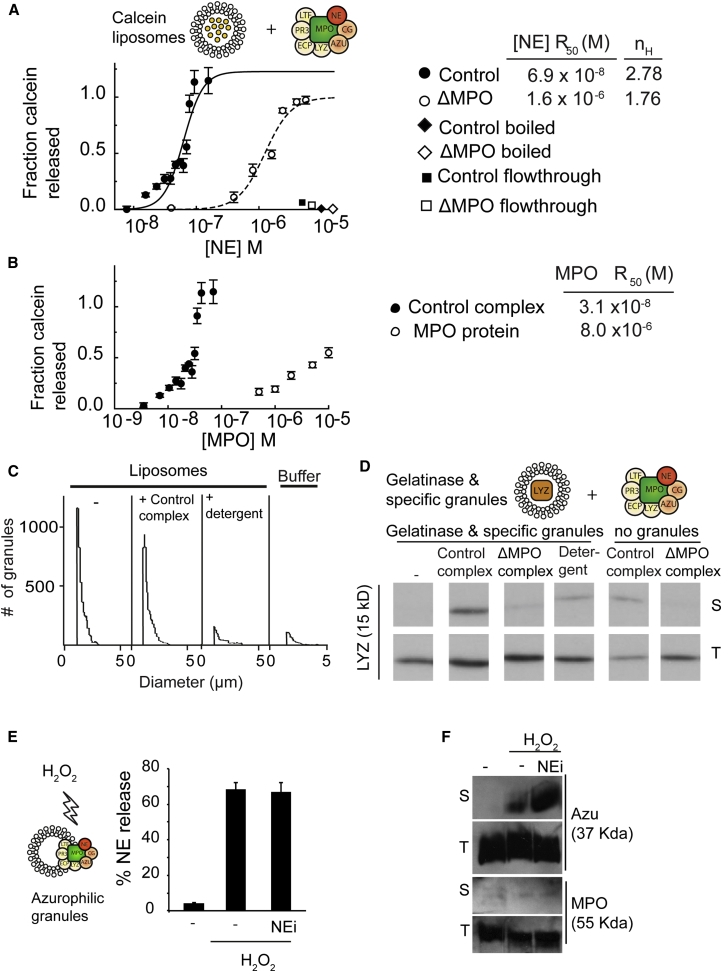
The Azurosome Promotes Translocation across Granule Membranes (A and B) Calcein release from synthetic PC/PS liposomes. Calcein fluorescence was measured after 15 min of incubation and normalized to liposomes alone and liposomes permeabilized with NP-40. Error bars indicate SD in duplicate samples. (A) Calcein release from synthetic liposomes incubated with control or ΔMPO azurosome, monitored by fluorescence dequenching of released calcein. The azurosome was quantified based on NE content as measured by ELISA and expressed in moles (x axis). Black and white squares: flowthrough buffer from the purification of control (black) and ΔMPO (white) azurosome. Black and white rhombuses: boiled samples (black) and ΔMPO azurosome (white) at the highest concentration. Fitting was used to calculate the concentration of azurosome required for 50% release (R_50_) and the apparent cooperativity coefficient (n_H_). (B) Calcein release from synthetic liposomes incubated with control azurosome or purified MPO, monitored by fluorescence. The azurosome was quantified based on MPO content as measured by ELISA and expressed in moles (x axis). Fitting was used to calculate the concentration of azurosome required for 50% release (R_50_). (C) CASY impedance cell counter analysis of calcein-loaded synthetic PC/PS liposomes, either untreated or incubated with azurosome from a control donor or NP-40. (D) Release of LYZ from specific and gelatinase granules incubated with control azurosome, ΔMPO azurosome, or NP-40. Samples were separated into soluble (S) and total (T) fractions by ultracentrifugation and immunoblotted against LYZ. Complexes without granules were used as controls for the background levels of LYZ from azurosomes alone. (E) NE release by azurophilic granules as it was captured and detected by NE ELISA. Duplicate reactions of intact azurophilic granules, untreated or treated with NEi and activated with 100 μM H_2_O_2_ for 30 min. Additional reactions in the same conditions but treated with NP-40 were used for total to calculate the fraction of NE released. Error bars indicate SD in duplicate samples. (F) AZU and MPO release from isolated native azurophilic granules alone or after incubation with H_2_O_2_ in the absence or presence of NEi. Samples were incubated for 30 min and insoluble granules were removed by centrifugation to yield soluble (S) protein. Total protein (T) prior to centrifugation. See also Figures S4 and S5.

Incubating the calcein-loaded liposomes with a purified control complex resulted in calcein release in a dose-dependent manner (Figure 5A). The data fitted to a sigmoidal dose-response curve with an apparent cooperativity Hill coefficient of 2.78, suggesting that approximately three complex molecules assemble on the membrane to mediate calcein release. Notably, azurosome isolated from control human donors released calcein approximately 40-fold more efficiently than azurosome isolated from a ΔMPO donor, indicating that MPO is critical for the release activity. Indeed, unlike azurocidin or NE (data not shown), purified MPO was sufficient to release calcein, albeit with 100-fold lower efficiency than the azurosome (Figure 5B). Preboiling the complex completely abrogated calcein release, indicating that the activity is mediated by proteins and not by residual detergent micelles (Figure 5A). The flowthrough from the azurosome purification did not induce release. We obtained similar calcein release curves with a control complex purified from azurophilic granules that were freeze-thawed and sonicated in the absence of detergent (Figure S5A). Impedance measurements confirmed that unlike the detergent control, the azurosome did not lyse liposomes (Figure 5C), indicating that it promotes release without rupturing or dissolving membranes. Adding purified MPO to the ΔMPO complex did not restore calcein release (Figure S5B), suggesting that a functional azurosome requires a particular assembly.

To examine whether the control azurosome was sufficient to mediate protein cargo release across native granule membranes, we incubated the complex with isolated specific and gelatinase native granules, which do not contain the complex, and monitored the release of LYZ from these granules into the soluble fraction after separation by ultracentrifugation (Figure 5D). The control azurosome mediated the release of significant levels of LYZ from native granules as compared with the low levels of the LYZ originating from the added control complex. In contrast, no LYZ was released by the ΔMPO complex, indicating that MPO is critical for the ability of the complex to translocate proteins across intact membranes. H_2_O_2_ activates the proteolytic activity of granule proteases in an MPO-dependent manner (Figure 2A). By testing fractions containing the purified azurosome, we found that the complex was sufficient to trigger protease activation (“posttreated,” Figure S3F). Interestingly, treatment with H_2_O_2_ or NEi did not affect calcein release (Figure S5C), suggesting that translocation is not regulated by oxidation, at least in vitro. In contrast, NE and AZU release from native azurophilic granules was dependent on H_2_O_2_ stimulation (Figures 5E and 5F). The release of these proteases from azurophilic granules was not blocked by NEi, indicating that oxidation promotes protease release by triggering NE dissociation from the complex without affecting the subsequent translocation across the membrane and without requiring NE activity during this step.

### NE Regulates F-actin Dynamics during Translocation to the Nucleus

**Figure 6:**
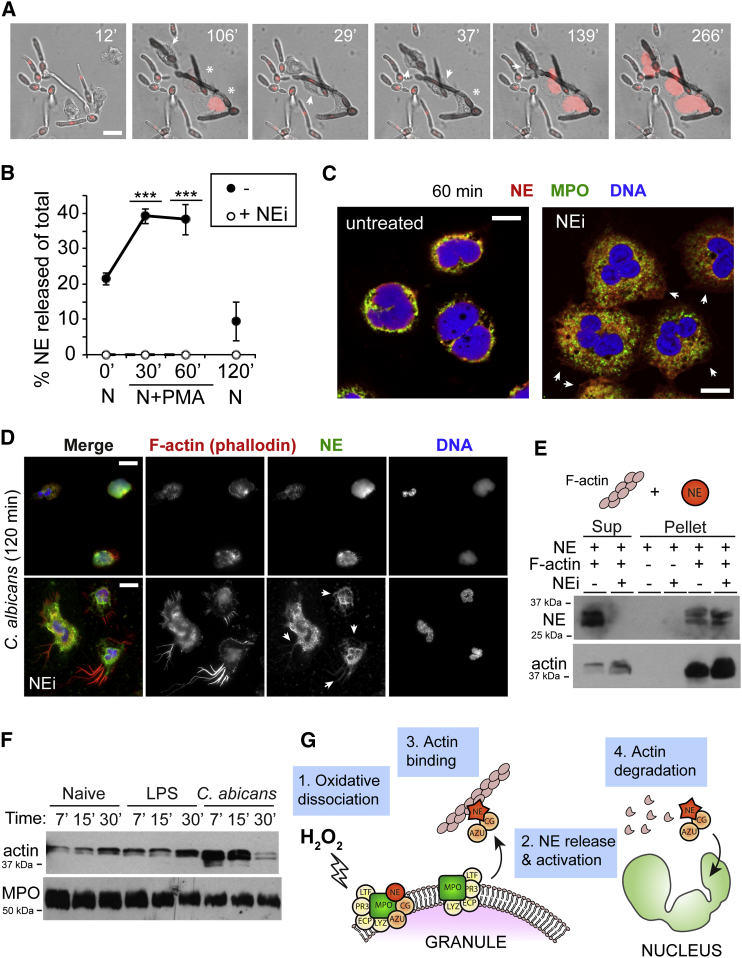
NE Regulates Actin Dynamics during Translocation to the Nucleus (A) Time lapse of live-cell microscopy depicting neutrophils depolarizing prior to the onset of nuclear decondensation in response to *C. albicans* (moi = 50). Scale bars, 10 μm. (B) NE release by control neutrophils either left naive or activated with PMA in the absence or presence of NEi (+NEi). ^∗∗∗^p < 0.001 comparing control versus NEi-treated at 30 min and 60 min. Error bars indicate SD in triplicate samples. (C) Untreated and NEi-treated neutrophils immunostained for NE (red), MPO (green), and DNA (DAPI, blue) 60 min after PMA stimulation. Arrows point to cytoplasmic areas containing NE in the absence of MPO. Scale bars, 5 μm. (D) Untreated and NEi-treated neutrophils immunostained for F-actin (phalloidin, red), NE (green), and DNA (DAPI, blue) 120 min after exposure to *C. albicans*. Arrows point to areas where NE and F-actin colocalize in the cytoplasm. Scale bars, 10 μm. (E) NE binding to F-actin in vitro by cosedimentation, showing NE alone or treated with NEi in the absence or presence of polymerized F-actin filaments. Reactions were incubated for 30 min at 37C and centrifuged at 100,000 *g* to generate supernatant containing soluble unbound protein supernatant (Sup) and actin-bound pellet. (F) Anti-Actin and anti-MPO immunoblots of whole-cell extracts of naive neutrophils or stimulated with LPS or *C. albicans* (moi = 10) at the indicated times after activation. (G) Mechanism of ROS-mediated NE translocation. In resting neutrophils, azurosome complexes are associated with a subset of azurophilic granule membranes. Upon oxidative activation (1), H_2_O_2_ triggers the release and activation of NE/CG/AZU protease complex into the cytoplasm (2). The complex binds to F-actin (3). The degradation of F-actin by active NE liberates the protease complex, allowing it to enter the nucleus.

Interestingly, during NETosis in response to *C. albicans*, the neutrophils depolarized and rounded up (Figure 6A; Movie S1). The timing of this global and abrupt downregulation of actin dynamics immediately prior to nuclear decondensation prompted us to ask whether it was linked to NE release. We previously reported that blocking NE activity prevents the translocation of NE to the nucleus (Papayannopoulos et al., 2010). To investigate NE localization in the presence of NEi, we isolated cytoplasm from activated neutrophils and cleared the granules and cytoskeleton by ultracentrifugation. We did not observe any soluble NE in the cytosol of activated neutrophils, suggesting that NE must be bound to an insoluble moiety (Figure 6B). Upon close examination of activated neutrophils, we observed that in the presence of NEi, NE failed to translocate to the nucleus, but localized in the cytoplasm away from azurophilic granules (Figure 6A). In contrast to untreated activated cells that appeared unpolarized, NEi-treated neutrophils remained polarized and continued to chemotax, indicating ongoing actin dynamics (Figures 6C and 6D). Strikingly, over time (2–3 hr), these neutrophils developed unusually large filopodia, where actin and NE colocalized (Figure 6D). This suggested that NE blocks actin dynamics and that inhibition of NE activity drives the accumulation of NE onto the actin cytoskeleton, preventing NE from reaching the nucleus. To test this hypothesis, we incubated purified NE with F-actin in the presence and absence of NEi and examined their association by an F-actin sedimentation assay. In the presence of NEi, purified NE bound to F-actin filaments in vitro and was sequestered to the actin pellet after cosedimentation occurred (Figure 6E). Strikingly, NE was present in the soluble fraction only in the absence of NEi, while some actin appeared degraded in the pellet. These data suggest that NE binds to F-actin in the cytoplasm and must degrade it in order to be free to translocate to the nucleus. Accordingly, in neutrophils undergoing NETosis in response to *C. albicans*, actin levels rapidly decreased by 30 min (the peak of NE translocation; Figure 6F), but not in response to soluble LPS, a weak inducer of NETosis. These findings expose an additional role of the azurosome in regulating actin dynamics through the modulation of NE proteolytic activity. This mechanism of arresting chemotaxis may also serve to deploy NETs in the vicinity of pathogens.

## Discussion

Our data suggest that resting neutrophils contain a subset of azurophilic granules in which specific antimicrobial proteins localize on the membrane (Figure 6G). Upon neutrophil stimulation, the oxidative burst generates H_2_O_2_ that triggers the activation and dissociation of NE, CG, and AZU from a complex that also contains MPO, LTF, PR3, and LYZ. We named this complex the “azurosome” because it was isolated from azurophilic granules. The detailed mechanism by which ROS promote dissociation remains unclear and may involve a reaction with MPO or other proteins of the complex. Importantly, MPO is required for the release of the proteases across intact membranes through a mechanism that remains to be elucidated. Once in the cytoplasm, NE binds the actin cytoskeleton and is sequestered in the insoluble fraction of the cytoplasm. The activation of NE by H_2_O_2_/MPO promotes F-actin degradation, liberating the protease to enter the nucleus.

Interestingly, the dissociation of the azurosome proteases is regulated by oxidation. The subsequent translocation of these proteases across the membrane is also mediated by the azurosome, which can mediate the release of other granule and liposome cargo constitutively in vitro. Our results suggest that protease dissociation is an active process, whereas crossing the membrane occurs passively. Translocation across the membrane via the azurosome may be bidirectional, but release may be driven entropically by a concentration gradient from the granule, where the cargo is highly concentrated, toward the cytosol, where cargo concentration is low. Interactions with the actin cytoskeleton and chromatin on the other side of the granule membrane may enhance this process thermodynamically. The proteolytic activity of NE may allow it to be liberated slowly from F-actin and enter the nucleus progressively to process histones and accumulate by binding tightly to the DNA. Accordingly, we find that during translocation, only 20%–40% of NE is soluble, indicating a slow transient process that is also reflected by immunofluorescence microscopy.

The ability of the purified azurosome to release native granule proteins without additional stimulation in vitro poses an interesting problem, since it suggests that granule cargo would be free to leak into the cytosol in naive neutrophils. As this is likely not the case, additional regulatory mechanisms may exist in vivo to prevent unregulated release. Notably, under naive conditions, granule proteins are thought to be packed in a semisolid state that may immobilize these proteins inside the granule and prevent interactions with the azurosome and subsequent translocation. However, more work is needed to address these issues.

The ability of the complex to release calcein and granule proteins when added exogenously to synthetic and native granules without disrupting membrane integrity (Figures 4A, 4C, and 4E) suggests that the azurosome incorporates into the lipid bilayer. The complex may assemble to form either a pore or protein transport machinery that transiently binds to cargo and rotates within the bilayer to release it on either side of the membrane. The cooperativity of the titration curve (Figure 4A) indicates that multiple azurosome molecules must assemble for efficient release. Multimerization is encountered in various pore-forming proteins and ion channels (Anderluh and Lakey, 2010), but it could also support a rotation model. Protein translocation across the membrane is dependent on MPO protein (Figure 4A), but not on MPO activity, as release of calcein and LYZ is greatly diminished in an MPO-deficient complex but is not affected by H_2_O_2_ stimulation (Figure 5C). Therefore, MPO is a key component that allows transport of cargo across membranes. MPO has also been shown to regulate neutrophil signaling via other nonenzymatic mechanisms during its activation of macrophage-1 antigen (Mac-1) (El Kebir and Filep, 2013).

Our experiments suggest that complex dissociation may not require the enzymatic activity of MPO either, since ABAH did not block dissociation and HOCl was not sufficient to promote it in vitro. However, these experiments should be considered with caution because ABAH does not completely block the generation of HOCl, and exogenously added HOCl may not be equivalent to the enzymatic product of MPO. Nevertheless, the enzymatic activity of MPO and its products play an important role in delivering NE to the nucleus, since the H_2_O_2_/MPO system activates the proteolytic activity of NE (Figure 2A). Although ABAH does not prevent NET formation, it slows down the process (Metzler et al., 2011), likely due to the delayed degradation of actin and histones. The azurosome-dependent protease activation highlights the importance of the complex as a protein scaffold that confers temporal and spatial specificity to oxidative signaling. ROS are highly reactive, short-lived molecules with low target specificity. The association of proteases with MPO allows the MPO/H_2_O_2_ system to specifically target the proteases for activation. ROS are known to regulate a wide range of cellular processes (Nathan, 2003; Tonks, 2005; Winterbourn, 2008) and this paradigm may operate in other signaling pathways. Inflammatory monocytes express MPO and are implicated in cardiovascular disease (Sugiyama et al., 2001). MPO may form complexes similar to the azurosome in these cells to regulate a variety of processes.

Our data show that NE drives nuclear decondensation but is also important for the disassembly of the actin cytoskeleton. This step may serve to immobilize neutrophils and allow the precise deployment of NETs within the site of infection. Furthermore, the dismantling of the cytoskeleton may serve to facilitate the disruption of the cytoplasmic membrane that precedes NET release. The degradation of actin may serve to reduce danger-associated actin signals during clearance of dying neutrophils by dendritic and scavenger cells (Ahrens et al., 2012). In addition, NE may degrade other proteins in the cytoplasm to affect additional neutrophil functions and the interaction with scavenging macrophages that regulate the resolution of inflammation (Serhan and Savill, 2005).

Our findings highlight the sophisticated architecture of neutrophil granules and suggest that distinct subsets of azurophilic granules may have different functions. Notably, azurosome-containing granules are not impermeable membrane compartments, but possess sophisticated machinery that mediates the selective, regulated release of granule cargo delivered through mechanisms that do not involve membrane fusion. The mechanism of NE release shares similarities with models that have been proposed to explain the release of cathepsins from intact lysosomes and the permeabilization of mitochondrial membranes during cell death (Boya and Kroemer, 2008). Bcl-2 proteins assemble on mitochondrial membranes and form pores that allow the release of molecules larger than 100 kDa without membrane rupture. Although some reports claim that these Bcl-2 proteins assemble on lysosomal membranes during apoptosis, it is not clear whether lysosomal protease release involves a protein transporter, a pore-forming complex, or lysis of a lysosome subpopulation. Importantly, ROS are important mediators of lysosomal cathepsin release during necrosis, suggesting that it may share functional similarities with NE release during NETosis.

We found that the azurosome is important in NETosis, but not during phagocytosis. This distinct role may be exploited for treating human disease. Inhibitors of the azurosome could potentially lead to therapies that inhibit NET formation specifically without disrupting other neutrophil functions.

## Experimental Procedures

### NET Formation

Human neutrophils were isolated from peripheral blood as previously described (Aga et al., 2002). We then seeded 5 × 10^4^ neutrophils per well in 24-well tissue culture plates in 1 ml RPMI with 10 mM HEPES and 1% fetal calf serum (FCS). Cells were allowed to settle at 37°C for 1 hr in the presence of a pharmacological inhibitor, when indicated, before stimulation with 100 nM PMA (Sigma-Aldrich). NETs were formed 2–4 hr after PMA or *C. albicans* (multiplicity of infection [moi] = 10) stimulation.

### Endogenous Histone Degradation in Activated Neutrophils

For each sample, three wells containing 2 × 10^5^ neutrophils were seeded in six-well plates in 3 ml RPMI, 10 mM HEPES, 1% FCS (for naive cells or PMA). Cells were stimulated with 100 nM PMA or plasma-opsonized *C. albicans* (moi = 10). At the indicated time points, the medium was removed and the cells were resuspended in 400 μl 1× Laemmli SDS buffer.

### Subcellular Fractionation

#### Preparation of Neutrophil Lysates

For experiments measuring NE release into the cytosol, 8 × 10^6^ neutrophils were seeded in 10 cm dishes in RPMI, 10 mM HEPES, and 1% FCS. They were allowed to settle for 30 min at 37°C in the absence or presence of 20 μM NEi (GW311616A; Sigma-Aldrich), 20 μM CG inhibitor (CGi, 219372; EMD), or 40 μg/ml PEG-catalase (C4963; Sigma-Aldrich), and then activated with 100 nM PMA. At the indicated time points, cells were scraped into 500 μl cold granule prep buffer (GPB) (20 mM HEPES pH 7.4, 100 mM KCl, 100 mM sucrose, 3 mM NaCl, 3 mM MgCl_2_, 1 mM EGTA). Naive cells were lysed by nitrogen cavitation at 400 psi for 2–3 min and the nuclei were removed by centrifugation at 300 × *g* for 15 min to generate low-speed supernatant (LSS). The LSS was centrifuged at 100,000 × *g* for 1 hr to yield high-speed supernatant (HSS).

#### Isolation of Granules

For granule preparations, LSS from 2–5 × 10^7^ neutrophils/ml was centrifuged (37,000 × *g*, 20 min) over a discontinuous (1.050, 1.090, and 1.120 g/ml) Percoll gradient as described previously (Kjeldsen et al., 1994; Lominadze et al., 2005).

### Enzymatic Assays

NE was quantitated using an ELISA kit (Hycult Biotechnology) and enzymatically by incubation with 300 μM Elastase Substrate I (MeOSuc-Ala-Ala-Pro-Val-pNA; Calbiochem). Concentrations of H_2_O_2_ and HOCl were measured as described previously (Morris, 1966; Noble and Gibson, 1970). MPO activity was measured with 0.1 mg/ml O-phenylenediamine (Sigma-Aldrich) in the presence of 500 μM H_2_O_2_.

### Protease Activity Assays

#### H4 Substrate

LSS extracts from naive or PMA-activated neutrophils were incubated for 3 hr with 5 μg/ml histone H4 (New England Biolabs) in the absence or presence of 0.2% NP-40 and then resolved by SDS-PAGE and immunoblotting against histone H4.

#### β-galactosidase Substrate

Purified β-galactosidase (10 U/ml; Sigma-Aldrich) was added to granules (30 μg/ml total protein) and incubated for 6–16 hr. Where noted, reactions included 0.2% NP-40 (labeled as “detergent”), 100 μM H_2_O_2_, 500 μM HOCl, 20 μM NEi, and/or 20 μM CGi. For colorimetric readout, 0.5 mg/ml X-gal (5-bromo-4-chloro-indolyl-β-D-galactopyranoside; Sigma-Aldrich) was added to the samples. For immunoblots, samples were dissolved in Laemmli SDS loading buffer.

### Azurosome Work

#### Immunoprecipitation

Azurophilic granules or LSS in GPB were left untreated or treated with 100 μΜ H_2_O_2_ for 2 hr at 37°C. Where indicated, granules were first treated with PIs (20 μM NEi, 20 μM CGi, 0.1 mM phenylmethanesulfonylfluoride [PMSF], or cOmplete Protease Inhibitor Tablet; Roche) or with the MPO inhibitor ABAH at 500 μM for 30 min on ice. The granules were then solubilized with 0.1% NP-40. Solubilized granules or LSS from 1 × 10^7^ neutrophils were incubated with 30 μg/ml rabbit anti-NE antibody (ab21595; Abcam) or mock antibody (rabbit anti-MMP9, A0150 [Dako] or rabbit anti-BPI, B2188 [Sigma]) for 2 hr at 4°C. Aliquots of total reactions were removed prior to antibody addition.

Protein-G Ultralink resin (Pierce) slurry (10–15 μl) was then added and incubated for 2 hr. Beads were rinsed three times in 1 ml GPB, three times in 1 ml GPB + 0.5 U/ml heparin to remove nonspecific ionic binding (Figure S2D), and then three times in 1 ml GPB. Bound proteins were eluted with 50 μl of 0.1 M glycine (pH 2.7) and then 10 μl of 1 M HEPES (pH 7.4) was added to neutralize the pH. Then 6× Laemmli sample buffer was added. Elutions and total reactions were boiled and analyzed by SDS-PAGE electrophoresis and Coomassie staining or immunoblotting.

#### Complex Purification

AZ granules, unactivated or preactivated with 100 μM H_2_O_2_ for 2 hr at 37°C, were permeabilized with 0.1% NP-40. Then 2 ml of sample was loaded onto a Superdex 200 GL column and eluted with 20 mM HEPES pH 7.4, 100 mM NaCl, plus 0.5 U/ml heparin to prevent nonspecific ionic binding (Figure S2D). For functional experiments, fractions containing the azurosome (usually 18–28) were combined and concentrated over Amicon Ultracell 3k filters to approximately 5–10 mg/ml total protein concentration.

### Protein Release

#### Release from Liposomes

Calcein-loaded liposomes were diluted into 200 μl osmo-PBS at 100 μM final total lipid concentration in the presence or absence of the indicated protein or azurosome concentrations. Where indicated, purified MPO (0.1–1 μM), 100 μM H_2_O_2_, or 10 μM NEi was added to the reactions. Samples were left to incubate for 10 min at 25°C and calcein fluorescence was recorded. Duplicate and triplicate samples were used for error calculation. After each read, 0.1% Triton X-100 was added to each sample to obtain the 100% permeabilization values. Data were normalized to the liposomes alone (lower limit) and liposomes in the presence of Triton (maximum) measurements.

#### Release from Granules

For release from granules, 15 μg of a mixture of specific and gelatinase granules, isolated as described above, was incubated with azurosome containing the equivalent of 50 nM NE (as measured by ELISA and semiquantitative immunoblot) in the absence or presence of 1% NP-40 for 1.5 hr at 37°C. An aliquot of the total reaction (T) was removed and reactions were centrifuged at 100,000 × *g* for 1 hr to yield the soluble fraction (S). Equivalent volumes of S and T fractions were dissolved in 1× Laemmli SDS loading buffer. The amount of LYZ or LTF in the azurosome alone control lanes was subtracted from the experimental lanes. The amount of LYZ or LTF in each soluble fraction was normalized to the total amount in the granules-alone sample.

#### Azurophilic Granule-Antibody Interaction

Rabbit anti-MPO and rabbit anti-BPI (0.5 μg) were mixed with freshly prepared azurophilic granules containing 30 μg total protein in 100 μl and incubated 30 min on ice. Reactions were overlaid over a 1.05, 1.09, and 1.12 mg/ml discontinuous Percoll gradient in 1× GPB and centrifuged at 37,000 × *g* for 20 min. The soluble supernatant was collected, upper Percoll layers were aspirated, and the 1.09/1.12 interface containing sedimented azurophilic granules was isolated. Then 5 μl of the soluble supernatant and 30 μl of the azurophilic fraction were analyzed by SDS-PAGE electrophoresis followed by western immunoblotting with a horseradish peroxidase-conjugated anti-rabbit immunoglobulin G (IgG) antibody.

#### Proteinase K Protection Assay

LSS or azurophilic granules prepared by nitrogen cavitation of 4 × 10^6^ neutrophils/ml in GPB were left untreated or pretreated with 0.2% Triton X-100 for 5 min at 37°C, and then left untreated or treated with 1–100 μg/ml proteinase K (Sigma-Aldrich) for 30 min at 37°C. After the reactions, 1 mM PMSF was added to inhibit proteinase K.

#### Degradation of S. flexneri IpaB by Neutrophils

*S. flexneri* M90T were cultured with 4.5 × 10^6^ neutrophils in RPMI-HEPES (moi = 30) in the absence or presence of 20 μM NEi and/or 500 μM of the MPO inhibitor ABAH. At 15 min postinfection, the medium was removed and cells were washed three times. At 90 min postinfection, the medium was removed and samples were lysed into 400 μl Laemmli SDS loading buffer.

#### Assessment of Granule Integrity Using the CASY Impedance Cell Counter

To assess granule integrity, 55 μg of azurophilic granules in 25 μl GPB, or 5 μg of PC/PS liposomes in 200 μl buffer was either left untreated or treated with 100 μM H_2_O_2_, 10 μg/ml purified azurosome, or 0.2%–0.5% NP-40 for 90 min at 37°C. Thereafter, reactions were diluted into PBS and impedance was measured using a CASY cell counter equipped with a 45 μM capillary.

#### Azurocidin Release

Azurophilic granules (50 μg total protein) were preincubated in the absence or presence of 20 μM NEi on ice for 30 min. H_2_O_2_ (100 μM) was added to 300 μl reactions and incubated for 30 min at 37°C. Then 50 μl of the reaction was removed for the total and the remaining reaction was centrifuged over a 1.050 g/ml Percoll layer at 37,000 × *g* for 20 min to remove granules and collect 50 μl of supernatant.

#### NE Release

Azurophilic granules in the presence of 10 μΜ NEi or vehicle (DMSO) were placed in human NE ELISA wells (Hycult Biotech) and stimulated with 100 μΜ H_2_O_2_ or 100 and 500 μΜ HOCl for 30 min at 37°C in GPB. Duplicate samples were used. For total samples, NP-40 was added prior to stimulation. Supernatants were removed, wells were washed with ELISA wash buffer, and NE protein released was detected with the ELISA kit.

#### F-actin Cosedimentation Assays

For F-actin cosedimentation assays, 0.5 mg/ml G-actin from rabbit muscle was polymerized for 1 hr at room temperature in 200 μl of 5 mM Tris-HCl pH 8.2, 50 mM KCl, 0.2 mM CaCl_2_, 1.2 mM ATP, 2 mM MgCl_2_, 0.5 mM dithiothreitol. Then 100 μl containing 0.3 mg/ml NE was centrifuged at 100,000 × *g* for 20 min at 4°C to remove aggregates. NEi was added to the supernatant where indicated and mixed with 200 μl polymerized actin, yielding a final NE concentration of 0.1 mg/ml. Reactions were incubated for 30 min at 37°C and centrifuged at 100,000 × *g* for 20 min at room temperature. The supernatant was carefully removed and the pellet was resuspended in 300 μl buffer.

#### Live-Cell Microscopy

Neutrophils were incubated with heat-inactivated *C. albicans* (moi = 50) in the presence of Sytox Green and imaged every 30 s for 4 hr by confocal microscopy (six frames per second).

## Author Contributions

K.D.M. and V.P. performed all experiments. A.L. performed actin-degradation experiments. C.G. stained and imaged electron micrographs. V.P. designed and directed the project. V.P., A.Z., and K.D.M. wrote the manuscript.

## Supplemental Information

Document S1. Supplemental Experimental Procedures and Figures S1–S5

Movie S1. Neutrophil Depolarization and Chemotactic Arrest during NETosis, Related to Figure 6 Time-lapse video of live-cell microscopy depicting neutrophils depolarizing while forming NETs in response to *C. albicans* (moi = 50) in the presence of the cell-impermeable dye Sytox Green, which stains DNA in permeabilized neutrophils. Phase-contrast and Sytox fluorescent images were obtained every 30 s for 4 hr by confocal microscopy. The movie was made at six frames per second.

Document S2. Article plus Supplemental Information
