# Supplementary material for: A Myeloperoxidase-Containing Complex Regulates Neutrophil Elastase Release and Actin Dynamics during NETosis
Source: Cell Rep. 2014 Jul 24;8(3):883–96. doi: 10.1016/j.celrep.2014.06.044 (PMC4471680; doi:10.1016/j.celrep.2014.06.044)
Supplement: Document S1. Supplemental Experimental Procedures and Figures S1–S5 [file mmc1.pdf]

Supplemental Figure 1

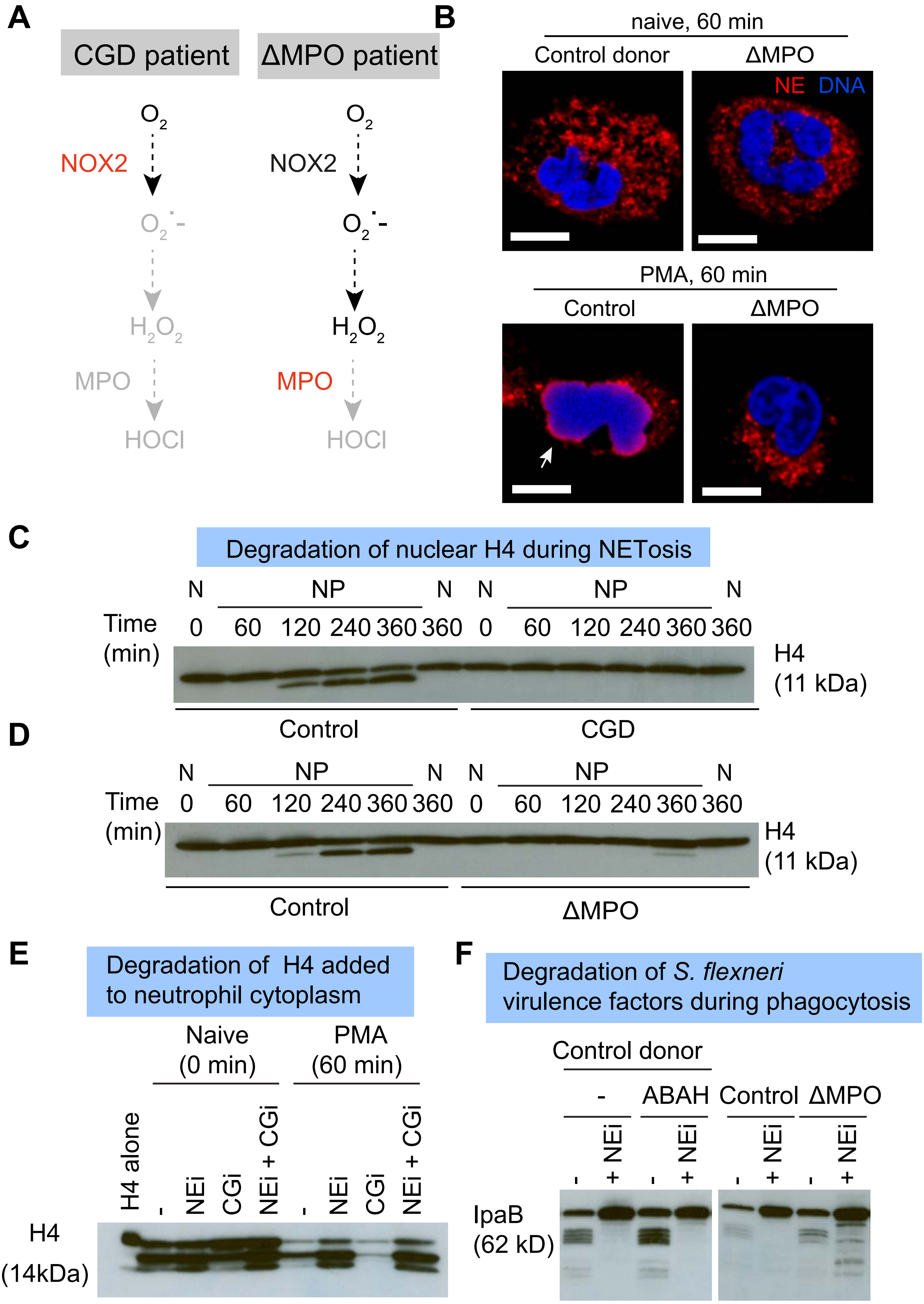

Supplemental Figure 1. ROS and MPO are required for NE translocation to the nucleus during NETosis, Related to Figure 1

(A) Simplified schematic of the reactive oxygen species (ROS) cascade in neutrophils and the defects (gray text) in ROS generation in chronic granulomatous disease (CGD) and ΔMPO neutrophils.
(B) Representative confocal immunofluorescence images of control donor and ΔMPO neutrophils, naïve or activated with PMA for 60 min, stained for DNA (blue) and immunolabeled for NE (red). Nuclear NE is shown with an arrow. Note the change in morphology of the decondensing nucleus. Scale bars: 5 μm
(C and D) Immunoblots against endogenous histone H4 in total cell lysates of neutrophils from control, CGD (C) and ΔMPO (D) donors, naïve or activated with PMA for the indicated durations.
(E) Degradation of exogenous histone H4 added to neutrophil cytoplasmic extract, naïve or activated for 60 min with PMA, in the absence or presence of NEi, CGi or both inhibitors. Cytoplasmic extracts were made by nitrogen cavitation, without detergent, to keep granule membranes intact.
(F) Digestion of IpaB by healthy donor (Control) and MPO-deficient (ΔMPO) neutrophils incubated with *S. flexneri* M90T at an MOI of 30 for 90 min, in the absence or presence of NEi and/or ABAH. IpaB degradation was assessed by immunoblotting.

Supplemental Figure 2

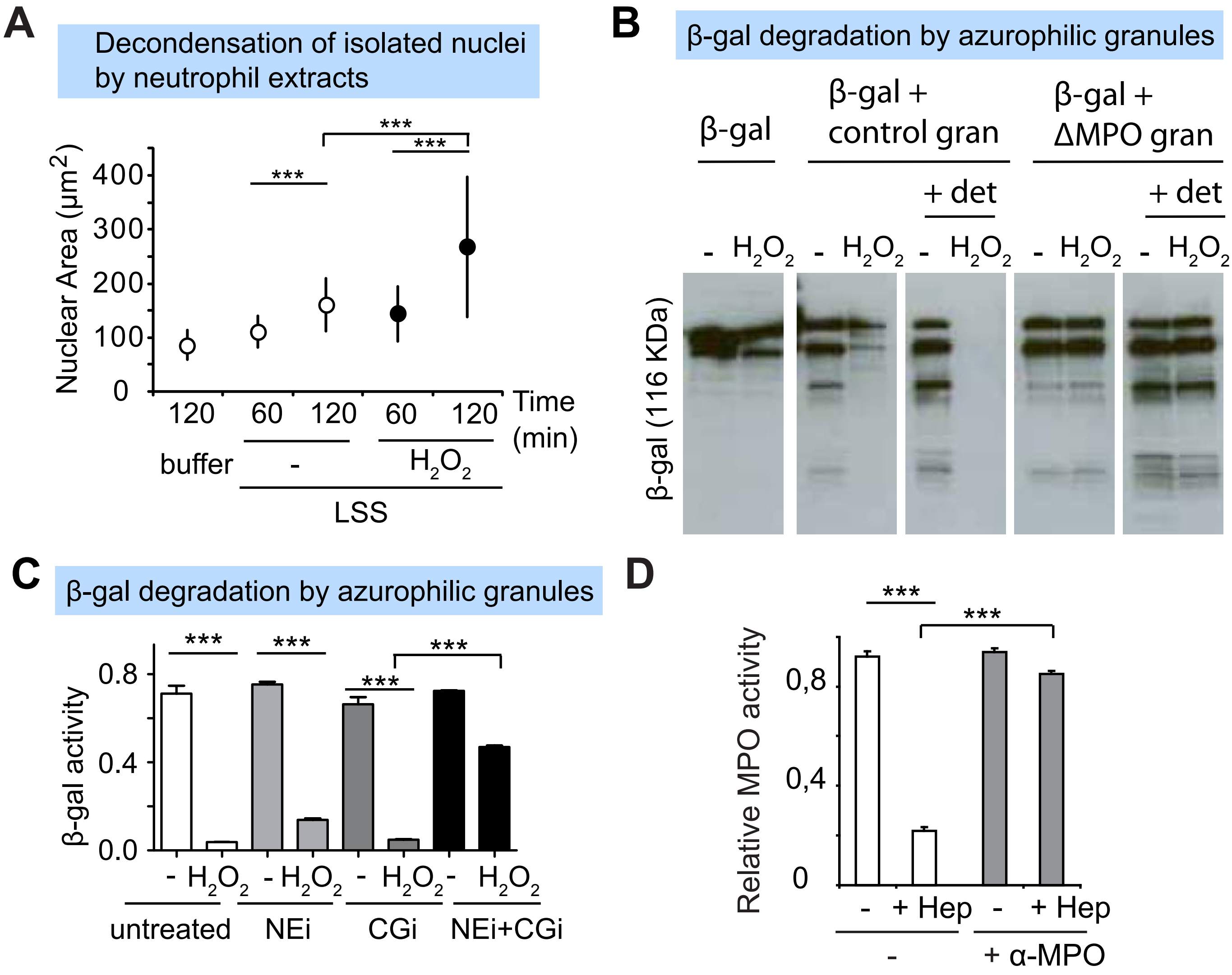

Supplemental Figure 2. H_2O_2 triggers NE release from azurophilic granules, Related to Figure 2

(A) Nuclear decondensation after treatment of isolated HL-60 nuclei with buffer or cytoplasmic extract (LSS) for the indicated durations, in the absence (white circles) or presence (black circles) of H_2O_2. Cytoplasmic extract was made by nitrogen cavitation, without detergent, to keep granule membranes intact. Nuclear decondensation was assessed by measuring Sytox-stained DNA areas from micrographs using ImageJ. ***: $P<0.001$, ANOVA: $P<0.0001$ analysis for the indicated group of samples.
(B) Degradation of β-galactosidase (β-gal) by azurophilic granules from control and ΔMPO neutrophils. The same samples from Figure 2A were assessed by immunoblotting against β-gal. Where indicated, H_2O_2 and/or NP-40 (det) were added to the reactions.
(C) β-gal activity against X-gal after incubation with azurophilic granules in the absence or presence of H2O2. Where indicated, granules were pre-incubated with NEi, CGi or both inhibitors. ***: $P<0.001$ between the indicated samples.
(D) Specific binding of MPO to Protein-G beads after washing with buffer containing heparin. MPO was incubated with Protein-G beads in the absence (white bars) or presence (gray bars) of anti-MPO IgG. Subsequently, samples were washed in the presence or absence of heparin (Hep). MPO bound to beads was detected by enzymatic activity against O-phenylenediamine. ***:

Supplemental Figure 3

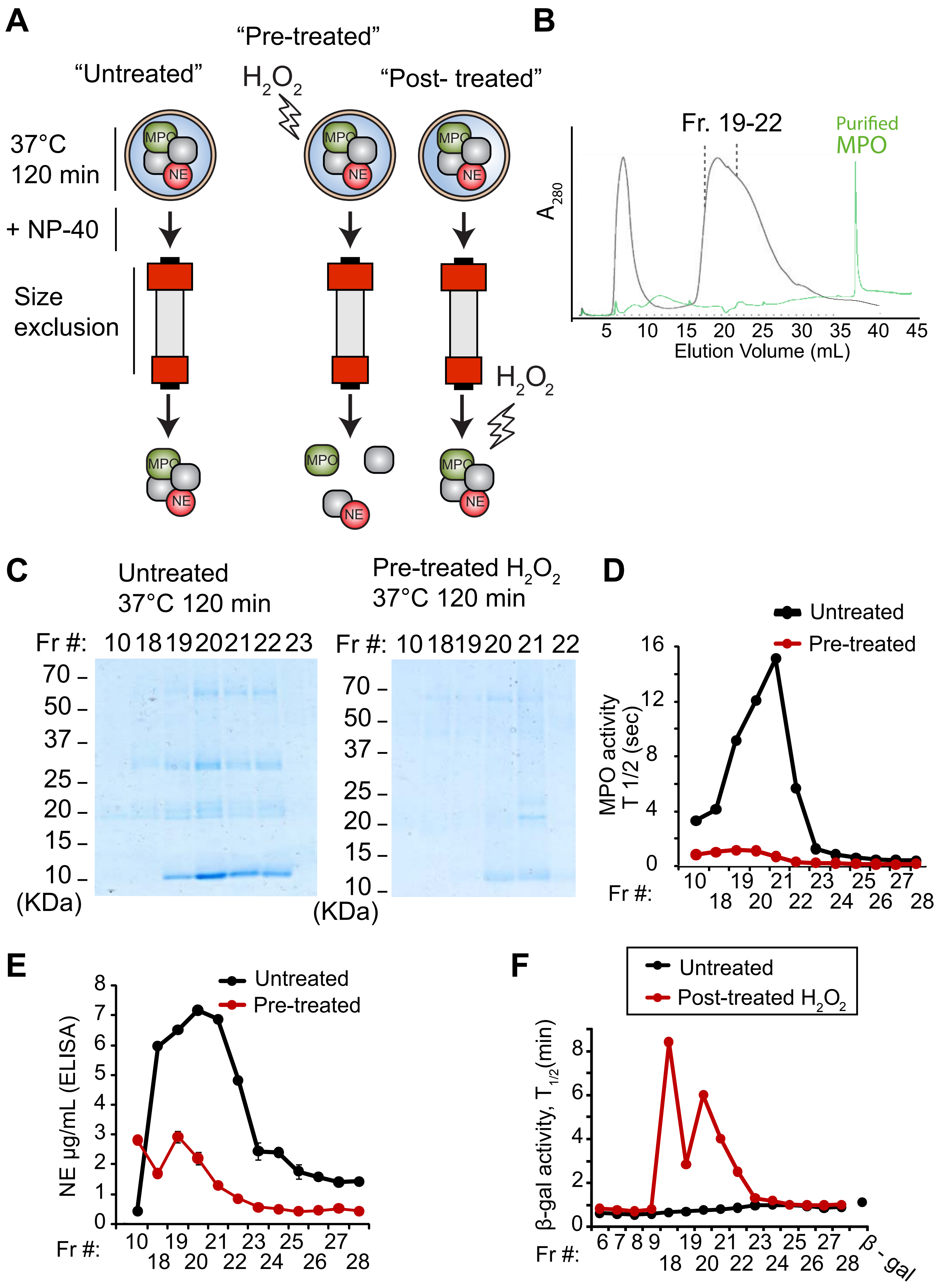

Supplemental Figure 3. Purification of the azurosome complex, related to Figure 2

(A) Schematic of azurosome purification by size exclusion chromatography. Untreated azurophilic granules were lysed with NP-40 and the soluble contents were purified over a Superdex 200 GL column. Pre-treated granules were stimulated for 120 min with H_2O_2, solubilized and passed over the column. Post-treated proteins were first purified by size exclusion chromatography from untreated granules and then were subsequently stimulated with H_2O_2 (Figure 6B).
(B) Size exclusion chromatography of azurophilic granules solubilized with NP-40 (black) or purified MPO (green) over a Superdex 200 GL column. Fractions 19-22 contain the azurosome.
(C) Coomassie stain of fractions from size exclusion of azurophilic granule extract, either untreated or pre-treated with H_2O_2. Protein abbreviations are the same as in Figure 2.
(D and E) Measurement of NE amount (D, by ELISA) and MPO activity (E, using chromogenic substrate) in fractions from untreated or H_2O_2-pre-treated granules.
(F) Protease activity against β-galactosidase of purified azurosome (same fractions as in Figure 2E), either untreated or post-treated with H_2O_2 (see cartoon in Figure S3A). Increase in proteolytic activity is plotted as a reduction in β-galactosidase activity, expressed as an increase in the $T_{1/2}$ of the enzymatic kinetic curve against X-gal.

Supplemental Figure 4

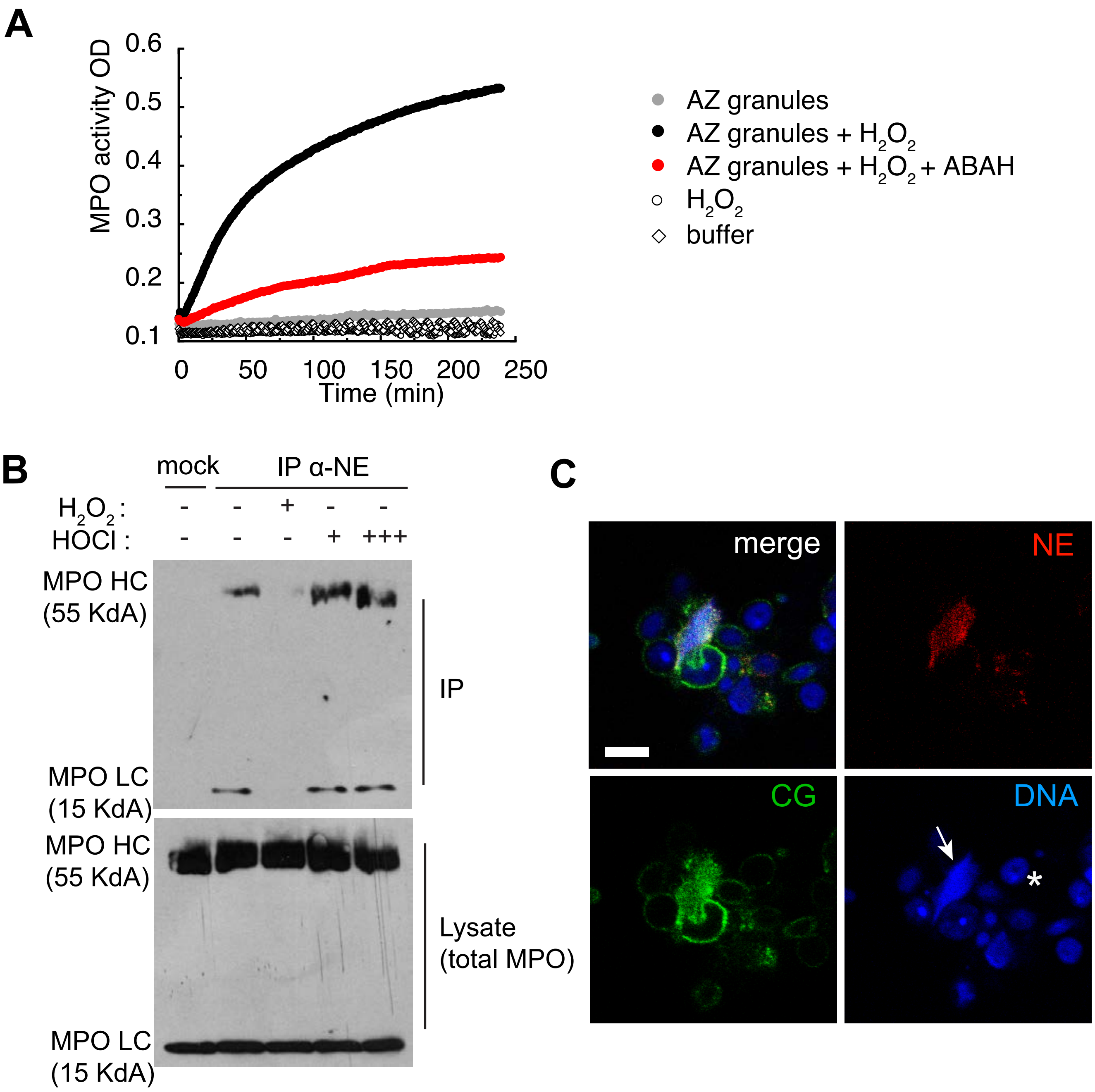

Supplemental Figure 4. Role of MPO enzymatic activity in the azurosome dissociation, Related to Figure 5

(A) MPO activity of azurophilic (AZ) granules by measuring O-phenylenediamine absorbance kinetics alone (grey circles) or in the presence of H2O2 (black circles) or inhibited by 500 µM ABAH.
(B) Immunoprecipitation with mock or anti-NE antibody of azurophilic granules after activation with 100 µM H_2O_2 or 100 µM or 500 µM HOCl for 2 hrs and solubilized with NP-40. Total reaction prior to immunoprecipitation shown (lower panel). Samples were immunoblotted for MPO.
(C) Immunostaining of neutrophils undergoing NETosis in response to C. albicans, showing co-translocation of NE (red) and CG (green) to the nucleus. Draq5 (DNA, blue). Neutrophil DNA (arrow), phagocytosed C. albicans DNA (asterisk). Scale bar: 5 µm.

Supplemental Figure 5

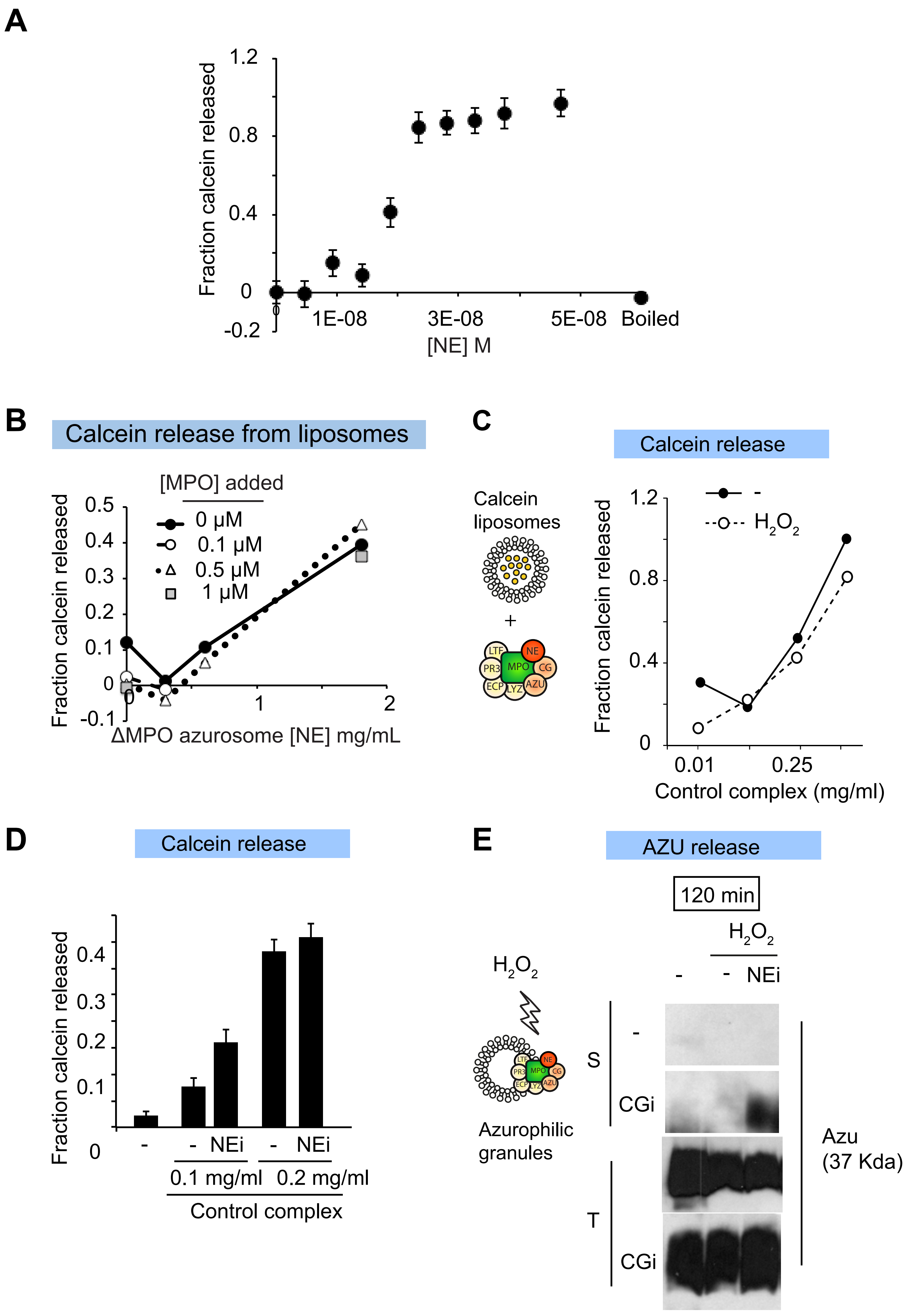

Supplemental Figure 5. Role of H_2O_2 and NE activity in protease release from granules, Related to Figure 5

(A) Calcein-loaded liposomes incubated with control complex purified from freeze-thawed and sonicated azurophilic granules that were not solubilized with detergent.
(B) Calcein release from liposomes incubated with increasing concentrations of azurosome from a ΔMPO donor. Where indicated, azurosome was pre-incubated with added purified MPO at the indicated concentrations.
(C) Liposomes were incubated with increasing concentrations of azurosome from a control donor, in the absence or presence of H_2O_2.
(D), Liposomes were incubated with azurosome from a control donor at the concentrations shown. Where indicated, azurosome was pre-incubated with NE inhibitor (NEi).
(E) AZU release from isolated native azurophilic granules alone or after incubation with H_2O_2 in the absence or presence of NEi or CGi (cathepsin G inhibitor. Samples were incubated for 120 min and insoluble granules were removed by centrifugation to yield soluble (S) protein. Total protein (T) prior to centrifugation. Under these experimental conditions, the released AZU was degraded at longer time points by activated NE and CG.

Supplemental Movie. Neutrophil depolarization and chemotactic arrest during NETosis, Related to Figure 6

Time-lapse of live cell microscopy depicting neutrophils depolarizing while forming NETs in response to *C. albicans* (MOI=50) in the presence of the cell impermeable dye Sytox Green that stains DNA in permeabilized neutrophils. Phase contrast and Sytox fluorescent images were obtained every 30 sec for 4 hrs by confocal microscopy. The movie was made at 6 frames per second.

Complete Experimental Procedures

Donors

All donors gave consent to blood drawing in accordance with the Declaration of Helsinki. Samples were collected with approval from the ethical committees at each institution. The ΔMPO donor bears a homozygous splice mutation (c.2031-2A>C/c.2031-2A>C, nomenclature according to (den Dunnen and Antonarakis, 2001))(Mauch et al., 2007), which generates null alleles. Lack of mature MPO protein and MPO activity was confirmed in (Metzler et al., 2011).

NET formation

Human neutrophils were isolated from peripheral blood as previously described (Aga et al., 2002). 5×10^4 neutrophils were seeded per well in 24-well tissue culture plates, in 1 ml RPMI with 10 mM HEPES and 1% FCS. Cells were allowed to settle at 37°C for 1h, in the presence of pharmacological inhibitor when indicated, before stimulation with 100 nM phorbol 12-myristate 13-acetate (PMA; Sigma-Aldrich). NETs were formed 2-4h after PMA stimulation.

Wild type Candida albicans was grown overnight at 30°C in YPD medium, which favors the yeast form of the organism. *C. albicans* were resuspended at 10x the desired multiplicity of infection (MOI=10) in 100% human plasma (obtained by centrifuging blood over Histopaque 1119 and collecting the upper layer) and incubated for 1h at room temperature. The mixture was diluted 1:10 into experimental sample wells and centrifuged for 5 min at 700 x *g* to synchronize the infection. NETs were formed within 2-4h after *C. albicans* stimulation.

Antibodies

Immunofluorescence staining

Neutrophils were fixed on glass coverslips using 2% paraformaldehyde (PFA). Samples were stained by permeabilizing with 0.2% Triton/PBS, blocking with 3% BSA and incubating for 1h with the following primary antibodies: mouse anti-neutrophil elastase (in-house, 1:200) and sheep anti-cathepsin G (Abcam ab8816, 1:200). Secondary antibodies conjugated to Cy2 and Cy3 were used (Jackson ImmunoResearch Laboratories, Inc., 1:300). DNA was stained with

Draq5 (1:300, Biostatus Limited) and actin with rhodamine-phalloidin (Invitrogen). Samples were imaged on a confocal fluorescence microscope (TCS SP-1; Leica) with a Fluotar 100× objective lens.

Immunoblotting

Protein samples on PVDF membranes were blocked with 3% BSA and probed using primary antibodies against: rabbit anti-histone H4 (Millipore 05-858, 1:5000); rabbit anti-MPO (polyclonal, Dako A0398, 1:10,000); goat anti-MPO heavy chain (Santa Cruz C-16, 1:1000); sheep anti-cathepsin G (Abcam ab8816, 1:1000); rabbit anti-azurocidin (Abcam ab67452, 1:1000); rabbit anti ß-galactosidase (Abcam ab4761, 1:1000); rabbit anti-bactericidal/permeability-increasing protein (BPI) (Sigma B2188, 1:1000); mouse anti-BPI (Abcam ab125121, 1:2000) or rabbit anti-lysozyme (Abcam ab91653, 1:1000). mouse anti-IpaB (a gift from PJ Sansonetti, Institut Pasteur, Paris, 1:1500); Secondary antibodies conjugated to horseradish peroxidase (Jackson) were used at a 1:20,000 dilution.

Endogenous histone degradation in activated neutrophils

For each sample, 3 wells containing 2 x 10^5 neutrophils each were seeded in 6-well plates, in 3 ml RPMI with 10 mM HEPES and 1% FCS (for naïve cells or when PMA was to be added), or without FCS but 3% human plasma for *C. albicans* experiments. Cells were allowed to settle at 37°C for 1h in the absence or presence of any inhibitors, before stimulation with 100 nM PMA or plasma-opsonized *C. albicans* at an MOI of 10. At the indicated time points, medium was removed and cells were resuspended in 400 µl total of 1X Laemmli SDS buffer, then samples were processed for immunoblotting against histone H4.

Subcellular fractionation

Preparation of neutrophil lysates

For experiments measuring NE release into cytosol, lysates were made from naïve or PMA-activated neutrophils from control or ΔMPO donors during a 2h timecourse. 8 x 10^6 neutrophils were seeded in 10 cm dishes in RPMI with 10

mM HEPES and 1% FCS, allowed to settle for 30 min at 37ºC in the absence or presence of 20 μM NE inhibitor (NEi, GW311616A, Sigma-Aldrich), 20 μM CG inhibitor (CGi, 219372, EMD), or 40 ug/ml PEG-catalase (C4963, Sigma-Aldrich), which can be taken up by endocytosis (Beckman et al., 1988), and activated with 100 nM PMA. At the indicated time points, cells were scraped into 500 μl cold granule prep buffer (GPB) (20 mM HEPES pH 7.4, 100 mM KCl, 100 mM sucrose, 3 mM NaCl, 3 mM $MgCl_2$, 1 mM EGTA). Naïve cells were lysed using nitrogen cavitation at 400 psi for 2-3 min(Udby and Borregaard, 2007) on ice; nuclei were removed by centrifugation at 300 x g for 15 min to generate LSS. LSS was centrifuged at 100,000 x g for 1h to yield high-speed supernatant. Measurements were taken in duplicates or triplicates and experiments were repeated 2-5 times.

Isolation of granules

For granule preparations, LSS from 2-5 x 10^7 neutrophils/ml was centrifuged (37,000 x *g*, 20 min) over a discontinuous (1.050, 1.090, and 1.120 g/ml) Percoll gradient as described previously (Kjeldsen et al., 1994; Lominadze et al., 2005).

Enzymatic assays

NE was quantitated using an ELISA kit (Hycult Biotechnology) according to the manufacturer's directions, or enzymatically by incubation with 300 mM Elastase Substrate I (MeOSuc-Ala-Ala-Pro-Val-pNA, Calbiochem) at 37°C and measuring endpoint absorbance at 410 nm. Concentrations of hydrogen peroxide (H_2O_2) and hypochlorous acid (HOCl) were measured as described in (Morris, 1966; Noble and Gibson, 1970). MPO activity was measured with 0.1 mg/ml O-phenylenediamine (Sigma-Aldrich) in the presence of 500 μM H_2O_2 at 25˚C; absorbance was monitored at 450 nm.

Protease activity assays

H4 substrate

LSS extracts from naïve or PMA-activated neutrophils were incubated for 3h with 5 μg/ml histone H4 (New England Biolabs) in the absence or presence of 0.2% NP-40, then resolved by SDS-PAGE and immunoblotting against histone H4.

β -galactosidase substrate

Purified β-galactosidase (β-gal, Sigma-Aldrich) (10 U/ml) was added to samples containing proteases in 96-well plates. Granules were used at 30 μg/ml total protein. Reactions were incubated for 6-16h, depending on the protease concentration, to allow proteases to degrade β-gal. Where noted, reactions included 0.2% NP-40 (labeled as "detergent"), 100 μM H_2O_2, 500 μM HOCl, 20 μM NEi and/or 20 μM CGi. For colorimetric readout, 0.5 mg/ml X-gal (5-bromo-4-chloro-indolyl-β-D-galactopyranoside, Sigma-Aldrich) was added to the samples and incubated for 30 min to 1h to develop blue color; absorbance was measured at 635 nm. Samples were measured in triplicate. For immunoblot readout, samples were dissolved in Laemmli SDS loading buffer, then processed for immunoblotting using an antibody against β -galactosidase.

Azurosome work

Evaluating non-specific binding to resins (Supplemental Figure 2D)

Heparin at 0.5 U/mL was used in washes in immunoprecipitation experiments and gel filtration to prevent non-specific ionic interactions of basic proteins with resins. This is a traditional approach used routinely in biochemical work with highly basic proteins. To evaluate the effectiveness of heparin in our system, purified 5 μM MPO alone or pre-incubated with 0.5 μg polyclonal rabbit anti-MPO (Dako A0398) was incubated with Protein-G Ultralink resin (Pierce) in PBS. Beads were washed 3X with PBS alone or in the presence of 0.5 U/mL heparin, followed by 3X with PBS alone. Beads were tested for MPO activity after 10 min incubation with 0.1 mg/ml O-phenylenediamine (Sigma-Aldrich) and 100 μM H_2O_2 and normalized to the activity of the initial amount of MPO added in the experiment.

Immunoprecipitation experiments

Azurophilic granules or LSS in GPB were left untreated or treated with 100 μM H_2O_2 for 2h at 37°C. Where indicated, granules were first treated with protease inhibitors (20 μM NEi, 20 μM CGi, 0.1 mM PMSF, and Complete protease inhibitor tablet (Roche)) or with the MPO inhibitor ABAH at 500 μM, for 30 min

on ice. Granules were then solubilized with 0.1% NP-40. Solubilized granules or LSS from $1x10^7$ neutrophils were incubated with 30 μg/mL rabbit anti-NE antibody (Abcam, ab21595) or mock antibody (rabbit anti-MMP9, Dako, A0150; or rabbit anti-BPI, Sigma, B2188) for 2h at 4ºC. Aliquots of total reactions were removed prior to antibody addition.

10-15 μL of Protein-G Ultralink resin (Pierce) slurry was added and incubated for 2h. Beads were rinsed 3 times in 1 mL GPB, 3 times in 1 mL GPB + 0.5 U/mL heparin, then 3 times in 1 mL GPB. Bound proteins were eluted with 50 μL 0.1 M glycine pH 2.7, then 10 μl 1M HEPES pH 7.4 was added to neutralize the pH. 6X Laemmli sample buffer was added; elutions and total reactions were boiled and analyzed by SDS-PAGE electrophoresis and Coomassie staining or immunoblotting.

The physiological concentration of H_2O_2 at the peak of the respiratory burst is difficult to measure. Theoretical calculations place it in the micromolar range. However, this is a continuous and sustained burst. We used 100 μM H_2O_2 in our *in vitro* experiments, to ensure that enough of the oxidant is present to saturate the system, but not at concentrations that would far exceed the theoretical physiological estimates. The levels of H_2O_2 used were in equivalent ranges to concentrations used in studies modeling metabolic oxidation (Anastasiou et al., 2011).

Complex purification

AZ granules, unactivated or pre-activated with 100 μM H_2O_2 for 2h at 37˚C, were permeabilized with 0.1% NP-40. 2 ml of sample was loaded onto a Superdex 200 GL column and eluted with 20 mM HEPES pH 7.4, 100 mM NaCl, plus 0.5 U/mL heparin to prevent non-specific ionic binding to the resin. For functional experiments, fractions containing the azurosome (usually 18-28) were combined and concentrated over Amicon Ultracell 3k filters to approximately 5-10 mg/mL total protein concentration.

Protein identification

Proteins were identified after in-gel tryptic digestions of excised bands as described in (Zimny-Arndt et al., 2009).

Protein release experiments

Preparation of calcein-filled synthetic liposomes

Phosphatidyl choline (egg yolk) and dipalmitoyl phosphatidyl-L-serine (brain) were purchased from Avanti Lipids. Large unilamellar PC:PS vesicles loaded with calcein were made by mixing lipids at a 4:1 molar ratio in chloroform, evaporating the chloroform over argon and rehydrating with PBS containing 50 mM calcein. Lipids were subjected to 10 rounds of flash freezing and thawing, followed by 1 min bath sonication. The liposomes were then centrifuged 1h at 100,000 x *g*, the supernatant was decanted and the pellet was reconstituted in PBS adjusted to 0.399 osmolarity with NaCl (osmo-PBS). This procedure was repeated 2 more times. Liposomes were resuspended in 200 µl osmo-PBS and extruded with a 0.4 µm filter.

Release from liposomes

This assay is based on the release of calcein stored at high concentration within synthetic liposomes. Calcein fluorescence is quenched inside the liposomes. Membrane permeabilization leads to release of calcein and a considerable increase in fluorescence. Calcein-loaded liposomes were diluted into 200 µL osmo-PBS at 100 µM final total lipid concentration in the presence or absence of the indicated protein or azurosome concentrations. Where indicated, purified MPO (0.1-1 µM), 100 µM H_2O_2, or 10 µM NEi were added to the reactions. Samples were left to incubate for 10 min at 25ºC and calcein fluorescence was recorded. Duplicate and triplicate samples were used for error calculation. After each read, 0.1% Triton X-100 was added to each sample to obtain the 100% permeabilization values. Data were normalized to the liposomes alone (lower limit) and liposomes in the presence of Triton (maximum) measurements. Samples were measured in triplicate.

Fitting release data

Permeabilization data were fitted by nonlinear squares methods using the Profit software and the equation $I = [Io + (Imax \times X^{(nH/Kd)}] / [1 + X^{(nH/Kd)}]$ where X

is the concentration of NE, I is the measured fluorescence intensity and Imax the fluorescence intensity at saturation, n_H is the Hill coefficient, and Kd is the apparent dissociation constant. Data are plotted as normalized fraction saturation Y where Y = (I - Io)/(Imax - Io) = $[X^{(nH/Kd)}]/[1 + X^{(nH/Kd)}]$. PC_{50}s were calculated as the concentrations required to obtain 50% permeabilization.

Release from granules

15 µg of a mixture of specific and gelatinase granules, isolated as described above, was incubated with azurosome containing the equivalent of 50 nM NE (as measured by ELISA and semi-quantitative immunoblot), in the absence or presence of 1% NP-40 for 1.5h at 37°C. An aliquot of total reaction (T) was removed and reactions were centrifuged at 100,000 x *g* for 1h to yield soluble fraction (S). Equivalent volumes of S and T fractions were dissolved in 1X Laemmli SDS loading buffer and resolved by SDS-PAGE, then analyzed by immunoblotting against LYZ or LTF.

Bands were quantified using the densitometry function in ImageJ. The amount of LYZ or LTF in the azurosome alone control lanes was subtracted from the experimental lanes. The amount of LYZ or LTF in each soluble fraction was normalized to the total amount in the granules alone sample.

Azurophilic granule-antibody interaction

Rabbit anti-MPO and rabbit anti-BPI (0.5 µg) was mixed with freshly prepared azurophilic granules containing 30 µg total protein in 100 µL and incubated 30 min on ice. Reactions were overlayed over a 1.05, 1.09 and 1.12 mg/mL discontinuous Percoll gradient in 1X granule prep buffer, and centrifuged at 37,000 x *g*, 20 min. The soluble supernatant was collected, upper Percol layers were aspirated and the 1.09 / 1.12 interface containing sedimented azurophilic granules was isolated. 5 µL of the soluble supernatant and 30 µL of the azurophilic fraction were analysed by SDS-PAGE electrophoresis followed by western immunoblotting with an HRP-conjugated anti-rabbit IgG antibody.

Proteinase K protection assay

LSS or azurophilic granules prepared by nitrogen cavitation of 4 x 10^6 neutrophils/ml in GPB was left untreated or pre-treated with 0.2% Triton X-100 for 5 min at 37˚C, then left untreated or treated with 1-100 µg/ml proteinase K (Sigma-Aldrich) for 30 min at 37˚C. After the reactions, 1 mM PMSF was added to inhibit proteinase K. Samples were dissolved in Laemmli SDS loading buffer and resolved by SDS-PAGE and immunoblotting against MPO and azurocidin.

Cryo-immunogold electron microscopy

For this analysis, 2 x 10^6 neutrophils were seeded per well in 6-well plates, in RPMI-HEPES with 1% FCS. At the indicated time points, cells were fixed with pre-warmed 2% PFA in PBS for 30 min at room temperature. The cells were harvested, gelatin-embedded and infiltrated with 2.3 M sucrose according to the method described(Peters PJ, 2006). Ultrathin sections were cut at -120°C with a RMC MTX/CRX cryo-ultramicrotome (Boeckeler Instruments Inc.), transferred to carbon- and pioloform-coated EM-grids and blocked with 1% bovine serum albumin, 0.02 M glycine and 10% cold water fish gelatin in PBS. The sections were incubated with appropriate dilutions in the same buffer of mouse monoclonal antibody directed against NE (NP57, Dako A/S Denmark) and rabbit polyclonal antibody directed against MPO (A0398, Dako A/S Denmark). Secondary antibody incubations were carried out with goat-anti-rabbit and goat-anti-mouse antibodies coupled to 12 nm and 6 nm gold particles (Jackson).

Specimens were then contrasted and embedded with uranyl-acetate/methyl-cellulose following the method described(Slot et al., 1991) and analyzed in a Leo 912AB or LEO 906 transmission electron microscope (Zeiss, Oberkochen, Germany) using a Cantega or Morada digital camera (Olympus Soft Imaging Solutions).

In vitro nuclear decondensation assays

As described in (Papayannopoulos et al., 2010). Briefly, low speed supernatant extracts containing soluble cytosolic proteins and granules were prepared from 5 × 10^7 neutrophils/ml, lysed by Nitrogen cavitation in GPB and 0.1 mM PMSF. Nuclei were removed by centrifugation at 300 g for 10 min. Intact HL60 nuclei were prepared from HL-60 cells differentiated with 5 µM retinoic acid for 96 h

and isolated as described by Celis (1998). Reactions were performed by mixing 10µl of neutrophil extract at 0.8 mg/ml total protein, with 10^4 nuclei and Sytox. 3-µl aliquots were transferred onto 12-well, 5-mm diagnostic slides (Menzel-Glaser) and covered with 20 × 50 mm coverslips. Reactions were performed in a humidified chamber at 37°C for the indicated timepoints in the absence or in the presence of 100µM H_2O_2. Decondensation of Sytox-labeled nuclei was documented by fluorescence microscopy and the area of each nucleus was calculated using ImageJ software.

Degradation of S. flexneri IpaB by neutrophils

Shigella flexneri M90T were cultured overnight at 37°C in TSB and subcultured 1:100 for 2 hours in fresh medium, washed in PBS and opsonized in 10% human serum in PBS for 30 min at 37°C, then washed in PBS and added to 4.5 x 10^6 cells in RPMI-HEPES at an MOI of 30 and centrifuged at 700 x g, in the absence or presence of 20 µM NEi and/or 500 µM of the MPO inhibitor ABAH (4-aminobenzoic acid hydrazide, Sigma-Aldrich). 15 minutes post-infection, media was removed and cells were washed three times. 90 minutes post-infection, media was removed and samples were lysed into 400 µl Laemmli SDS loading buffer.

Assessment of granule integrity using CASY impedance cell counter

55 µg of azurophilic granules in 25 µl GPB, or 5 µg of PC/PS liposomes in 200 µl buffer, were either left untreated or treated with 100 µM H_2O_2, 10 µg/ml purified azurosome or 0.2-0.5% NP-40 for 90 min at 37˚C. Thereafter, reactions were diluted into PBS and impedance was measured using a CASY cell counter equipped with a 45 µM capillary.

Azurocidin release

Azurophilic granules (50 µg total protein) were pre-incubated in the absence or presence of 20 µM NEi on ice for 30 min. 100 µM H_2O_2 was added to 300 µL reactions and incubated for 30 min at 37˚C. 50 µL of the reaction were removed for the total and the remaing was centrifuged over a 1.050 g/mL Percoll layer at 37,000 x *g*, 20 min to remove granules and collect 50 µL of supernatant.

NE release

Azurophilic granules in the presence of 10 μM NEi, or vehicle (DMSO) were placed in human NE ELISA wells (Hycult Biotech) and stimulated with 100 μM H_2O_2 or 100 and 500 μM HOCl for 30 min at 37°C in granule prep buffer. Duplicate samples were used. For total samples, NP-40 was added prior to stimulation. Supernatants were removed, wells were washed with ELISA wash buffer and NE protein released was detected with the ELISA kit.

F-Actin co-sedimentation assays

0.5 mg/mL G-actin from rabbit muscle was polymerized for 1 hr at RT in 200μL of 5 mM Tris-HCl pH 8.2, 50mM KCl, 0.2 mM $CaCl_2$, 1.2 mM ATP, 2 mM $MgCl_2$, 0.5 mM DTT. 100μL containing 0.3 mg/mL NE was centrifuged at 100,000 x *g* for 20 min at 4°C to remove aggregates. NEi was added to supernatant where indicated and mixed with 200 μL polymerized actin yielding a final NE concentration of 0.1 mg/mL. Reactions were incubated for 30 min at 37°C and centrifuged at 100,000 x *g, 20 min, RT*. The supernatant was carefully removed and the pellet was resuspended in 300 μL buffer.

Live cell microscopy

Neutrophils were incubated with heat-inactivated *C. albicans* (MOI=50) in the presence of the cell impermeable dye Sytox Green that stains DNA in permeabilized neutrophils. Phase contrast and Sytox fluorescent images were obtained every 30 sec for 4 hrs by confocal microscopy. The movie was made at 6 frames per second.

Statistical analysis

All experiments were repeated 2-5 times and representative results are shown. In Figure S2A, raw measurements were analyzed in GraphPad Prism 5 software using the Kruskal-Wallis analysis of variance and Dunn multiple comparison test. All other measurements were analyzed in Microsoft Excel by unpaired T-test.

Experimental Procedure References

Aga, E., Katschinski, D.M., van Zandbergen, G., Laufs, H., Hansen, B., Muller, K., Solbach, W., and Laskay, T. (2002). Inhibition of the spontaneous apoptosis of neutrophil granulocytes by the intracellular parasite Leishmania major. J Immunol *169*, 898-905.

Anastasiou, D., Poulogiannis, G., Asara, J.M., Boxer, M.B., Jiang, J.K., Shen, M., Bellinger, G., Sasaki, A.T., Locasale, J.W., Auld, D.S*., et al.* (2011). Inhibition of pyruvate kinase M2 by reactive oxygen species contributes to cellular antioxidant responses. Science *334*, 1278-1283.

Beckman, J.S., Minor, R.L., Jr., White, C.W., Repine, J.E., Rosen, G.M., and Freeman, B.A. (1988). Superoxide dismutase and catalase conjugated to polyethylene glycol increases endothelial enzyme activity and oxidant resistance. J Biol Chem *263*, 6884-6892.

den Dunnen, J.T., and Antonarakis, E. (2001). Nomenclature for the description of human sequence variations. Human Genetics *109*, 121-124.

Kjeldsen, L., Sengelov, H., Lollike, K., Nielsen, M.H., and Borregaard, N. (1994). Isolation and characterization of gelatinase granules from human neutrophils. Blood *83*, 1640-1649.

Lominadze, G., Powell, D.W., Luerman, G.C., Link, A.J., Ward, R.A., and McLeish, K.R. (2005). Proteomic analysis of human neutrophil granules. Molecular & Cellular Proteomics *4*, 1503-1521.

Mauch, L., Lun, A., O'Gorman, M.R.G., Harris, J.S., Schulze, I., Zychlinsky, A., Fuchs, T., Oelschlagel, U., Brenner, S., Kutter, D*., et al.* (2007). Chronic granulomatous disease (CGD) and complete myeloperoxidase deficiency both yield strongly reduced dihydrorhodamine 123 test signals but can be easily discerned in routine testing for CGD. Clinical Chemistry *53*, 890-896.

Metzler, K.D., Fuchs, T.A., Nauseef, W.M., Reumaux, D., Roesler, J., Schulze, I., Wahn, V., Papayannopoulos, V., and Zychlinsky, A. (2011). Myeloperoxidase is required for neutrophil extracellular trap formation: implications for innate immunity. Blood *117*, 953-959.

Morris, J.C. (1966). Acid ionization constant of HOCl from 5 to 35 degrees. Journal of Physical Chemistry *70*, 3798-&.

Noble, R.W., and Gibson, Q.H. (1970). Reaction of ferrous horseradish peroxidase with hydrogen peroxide. Journal of Biological Chemistry *245*, 2409-&.

Papayannopoulos, V., Metzler, K.D., Hakkim, A., and Zychlinsky, A. (2010). Neutrophil elastase and myeloperoxidase regulate the formation of neutrophil extracellular traps. Journal of Cell Biology *191*, 677-691.

Peters PJ, B.E.a.G.A. (2006). Cryo-immunogold electron microscopy. In Current Protocols in Cell Biology (John Wiley & Sons, Inc.), pp. 4.7.1-4.7.19.

Slot, J.W., Geuze, H.J., Gigengack, S., Lienhard, G.E., and James, D.E. (1991). Immuno-localization of the insulin regulatable glucose transporter in brown adipose-tissue of the rat. Journal of Cell Biology *113*, 123-135.

Udby, L., and Borregaard, N. (2007). Subcellular fractionation of human neutrophils and analysis of subcellular markers. In Methods in Molecular Biology, M.T.D.F.R.B.G.M. Quinn, ed., p. 35.

Zimny-Arndt, U., Schmid, M., Ackermann, R., and Jungblut, P.R. (2009). Classical proteomics: two-dimensional electrophoresis/MALDI mass spectrometry. Methods Mol Biol *492*, 65-91.
