# Supplementary figures and images for: A Myeloperoxidase-Containing Complex Regulates Neutrophil Elastase Release and Actin Dynamics during NETosis
Source: Cell Rep. 2014 Jul 24;8(3):883–96. doi: 10.1016/j.celrep.2014.06.044 (PMC4471680; doi:10.1016/j.celrep.2014.06.044)

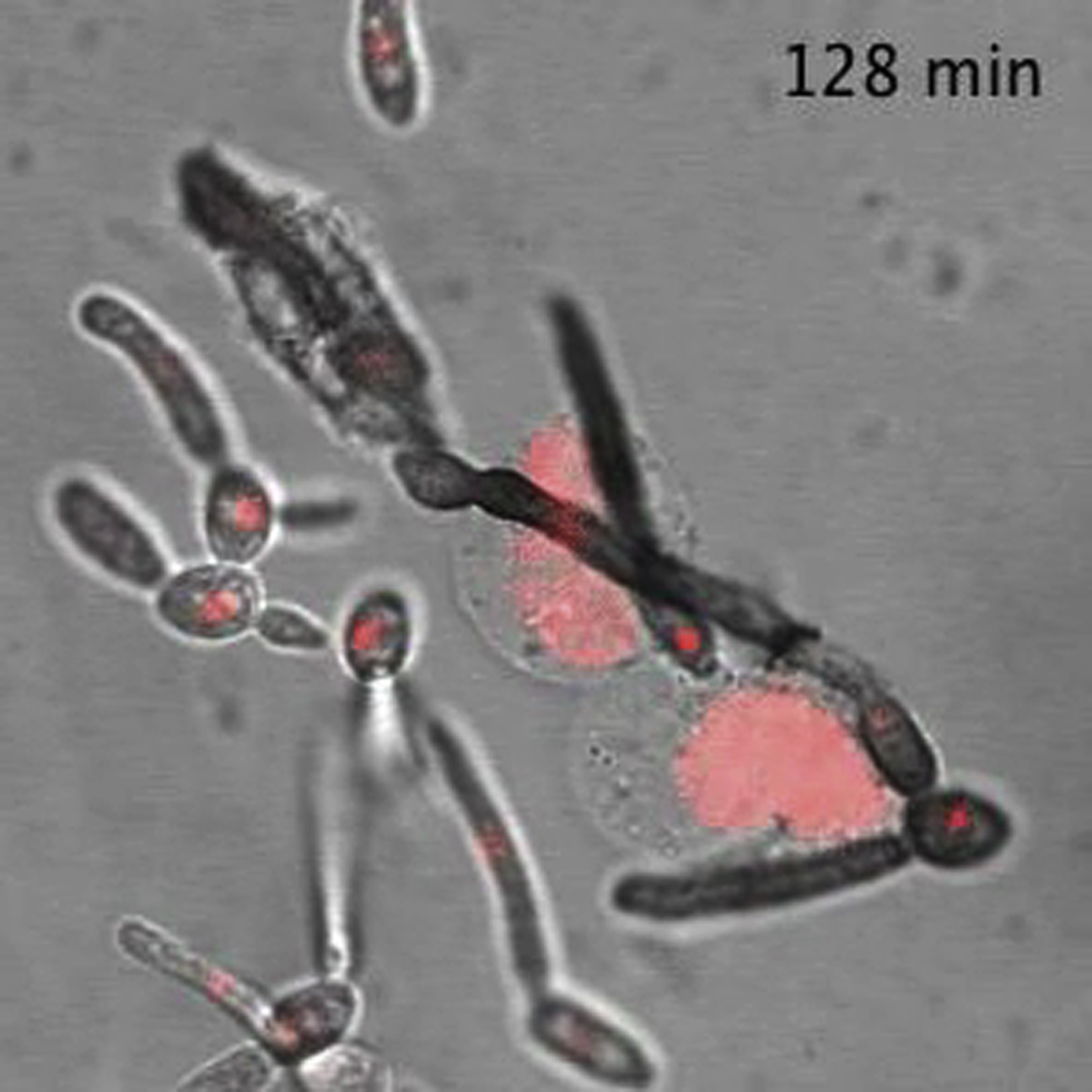

Supplement: Movie S1. Neutrophil Depolarization and Chemotactic Arrest during NETosis, Related to Figure 6 — Time-lapse video of live-cell microscopy depicting neutrophils depolarizing while forming NETs in response to C. albicans (moi = 50) in the presence of the cell-impermeable dye Sytox Green, which stains DNA in permeabilized neutrophils. Phase-contrast and Sytox fluorescent images were obtained every 30 s for 4 hr by confocal microscopy. The movie was made at six frames per second. [file mmc2.jpg]
